# SARS-CoV-2 Targets by the pscRNA Profiling of ACE2, TMPRSS2 and Furin Proteases

**DOI:** 10.1016/j.isci.2020.101744

**Published:** 2020-10-28

**Authors:** Lulin Zhou, Zubiao Niu, Xiaoyi Jiang, Zhengrong Zhang, You Zheng, Zhongyi Wang, Yichao Zhu, Lihua Gao, Hongyan Huang, Xiaoning Wang, Qiang Sun

**Affiliations:** 1 Institute of Biotechnology, 20 Dongda Street, Beijing 100071, P.R. China; 2 School of Medicine, Nankai University, 94 Weijin Road, Tianjin 300071, P. R. China; 3 Department of Oncology, Beijing Shijitan Hospital of Capital Medical University, 10 TIEYI Road, Beijing 100038, P. R. China; 4 School of Laboratory Medicine and Biotechnology, Southern Medical University, Guangzhou 510515, P. R. China; 5 National Clinic Center of Geriatric, the Chinese PLA General Hospital, Beijing 100853, P. R. China

**Keywords:** Virology, Microbiology, Transcriptomics

## Abstract

The cellular targets of SARS-CoV-2, the novel coronavirus causing the COVID-19 pandemic, is still rudimentary. Here, we incorporated the protein information to analyze the expression of ACE2, the SARS-CoV-2 receptor, together with co-factors, TMPRSS2 and Furin, at single-cell level *in situ*, which we called protein-proofed single-cell RNA (pscRNA) profiling. Systemic analysis across 36 tissues revealed a rank list of candidate cells potentially vulnerable to SARS-CoV-2. The top targets are lung AT2 cells and macrophages, then cardiomyocytes and adrenal gland stromal cells, followed by stromal cells in testis, ovary, and thyroid, whereas the kidney proximal tubule cells, cholangiocytes, and enterocytes are less likely to be the primary SARS-CoV-2 targets. Actually, the stomach may constitute a physical barrier against SARS-CoV-2 as the acidic environment (pH < 2.0) could completely inactivate SARS-CoV-2 pseudo-viruses. Together, we provide a comprehensive view on the potential SARS-CoV-2 targets by pscRNA profiling.

## Highlights

• A novel method of protein-proofed single-cell RNA (pscRNA) profiling was developed
• A rank list of 10 potential SARS-CoV-2 target cells were proposed
• The intestinal enterocytes are less likely to be the primary SARS-CoV-2 targets

## Graphical Abstract

**Figure:**
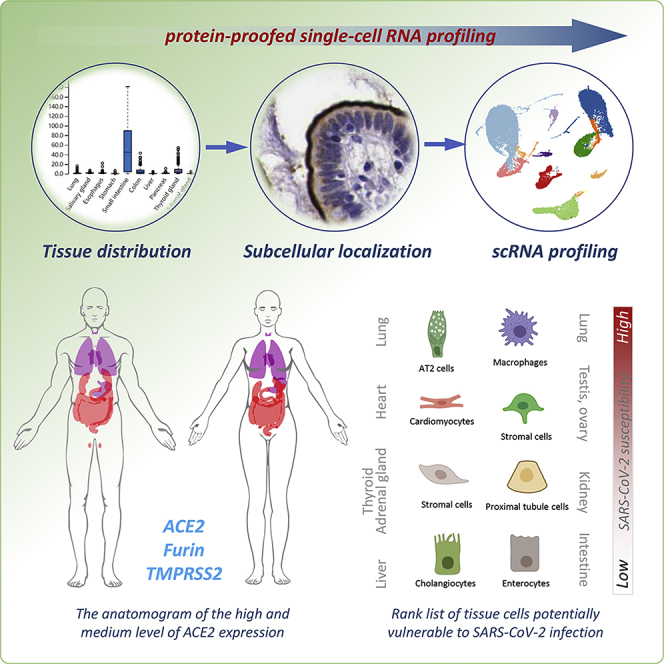

## Introduction

In January, 2020, a novel coronavirus of unknown origin was identified to cause severe pneumonia in about 15%–20% infected patients (Guan et al., 2020; Novel Coronavirus Pneumonia Emergency Response Epidemiology, 2020); the disease is currently called COVID-19 abbreviated from coronavirus disease (2019) (Zhu et al., 2020). The virus is phylogenetically similar (∼76% amino acid identity) to the severe acute respiratory syndrome coronavirus (SARS-CoV) (Tang et al., 2020; Wu et al., 2020b; Xu et al., 2020; Zhou et al., 2020b; Zhu et al., 2020) and was subsequently named as SARS-CoV-2 after the initial name of 2019-nCoV. The SARS-CoV-2 virus, particularly the G614 line (Jiang et al., 2020; Korber et al., 2020), is much more contagious than SARS-CoV, and had infected more than 40 million individuals from 215 countries and territories as of October 20, 2020, leading to more than 111,000 deaths with an average motility rate of about 4.5% (WHO, 2020). The pandemic of COVID-19 is posing a global health emergency.

The coronavirus is a large group of enveloped, single-strand positive-sense RNA viruses, with SARS-CoV and Middle East respiratory syndrome coronavirus (MERS-CoV) the two known deadly viruses for humans (Li, 2016). The spike (S) envelope glycoproteins on coronavirus are the major determinants of host cell entry. Proteolytic cleavage of S protein produces S1, the N-terminal region of S protein that is responsible for receptor binding, and S2, the trans-membrane C-terminal region of S protein that promotes membrane fusion. The cleavage step is often permissive for the fusion function of S protein as it helps to release the fusion peptide to insert into the target cellular membrane (Li, 2016; Millet et al., 2015). Therefore, the host range and cell/tissue tropism of coronaviruses were believed to be controlled by the S protein engagement of host cell receptor, and by the proteolytic cleavage of the S protein as well (Millet et al., 2015). Recently, works from several groups demonstrated, either bioinformatically or experimentally, that angiotensin-converting enzyme 2 (ACE2), the receptor for SARS-CoV virus (Li et al., 2003), is also a functional cellular receptor for SARS-CoV-2 virus (Hoffmann et al., 2020; Walls et al., 2020; Wrapp et al., 2020; Xu et al., 2020; Zhou et al., 2020b), and transmembrane protease serine 2 (TMPRSS2) and Furin are two proteases that process SARS-CoV-2 S protein to establish efficient infection (Hoffmann et al., 2020; Li et al., 2020; Meng et al., 2020; Walls et al., 2020).

Single-cell RNA (scRNA) profiling is a state-of-the-art tool to dissect gene expression at the single-cell level, and therefore was employed to explore the target cells of the SARS-CoV-2. Based on the profiling of ACE2 mRNA expression in different tissues/organs, multiple types of cells were proposed to be potentially targeted by SARS-CoV-2 virus, including the lung alveolar type 2 (AT2) cells (Zhao et al., 2020), nasal epithelial cells (WU et al., 2020a), esophageal epithelial cells and intestinal enterocytes (Zhang et al., 2020), liver cholangiocytes (Chai et al., 2020), cardiomyocytes (Xin Zou et al., 2020), kidney proximal tubule cells (Lin et al., 2020), and spermatogonia and Leydig/sertoli cells in the testis (Zhengpin et al., 2020). However, unparallel to the many potential targets cells and organs proposed, the COVID-19 patients primarily displayed typical symptoms of inflammation in the lung, where only a very small portion of cells (∼0.64% of total cells and ∼1.4% of AT2 cells) expressed ACE2 mRNA (Zhao et al., 2020); meanwhile, the injuries in other organs/tissues, such as the kidney and the intestinal track, where ACE2 gene was expressed at high levels, seemed to be uncommon (Guan et al., 2020; Wang et al., 2020). This obvious discrepancy suggests that mechanisms other than ACE2 mRNA levels are also involved in the regulation of SARS-CoV-2 infection of their target cells.

Considering that mRNA level does not always dictate comparable protein expression and subcellular localizations, which are missing information from mRNA profiling, are critical for protein functions, we set out to explore ACE2 expression at both mRNA and protein levels by taking advantages of the curated public database. We called this method as protein-proofed scRNA (pscRNA) profiling. Moreover, we also analyzed the co-expression of ACE2 with its two processing proteases, TMPRSS2 and Furin, at single-cell resolution *in situ* by pscRNA profiling. Systemic analysis of 36 human tissues/organs revealed the following. (1) A rank list of potential SARS-CoV-2 targets with lung AT2 cell and macrophages as the top targets, then cardiomyocytes, and then stromal cells in testis, ovary, adrenal, and thyroid glands. Among them, the lung macrophages and the stromal cells in ovary and adrenal gland were identified for the first time, which may account for severe clinical symptoms and rapid disease progression. (2) The mRNA levels may differ dramatically from the protein levels for ACE2, TMPRSS2, and Furin in different tissue cells, and protein subcellular localization is another factor potentially affecting virus host entry. (3) The co-expression of ACE2 with TMPRSS2 and Furin proteases may contribute to establish efficient infection of SARS-CoV-2 virus.

## Results

### Tissue Distribution of ACE2, TMPRSS2, and Furin Proteases

**Figure 1:**
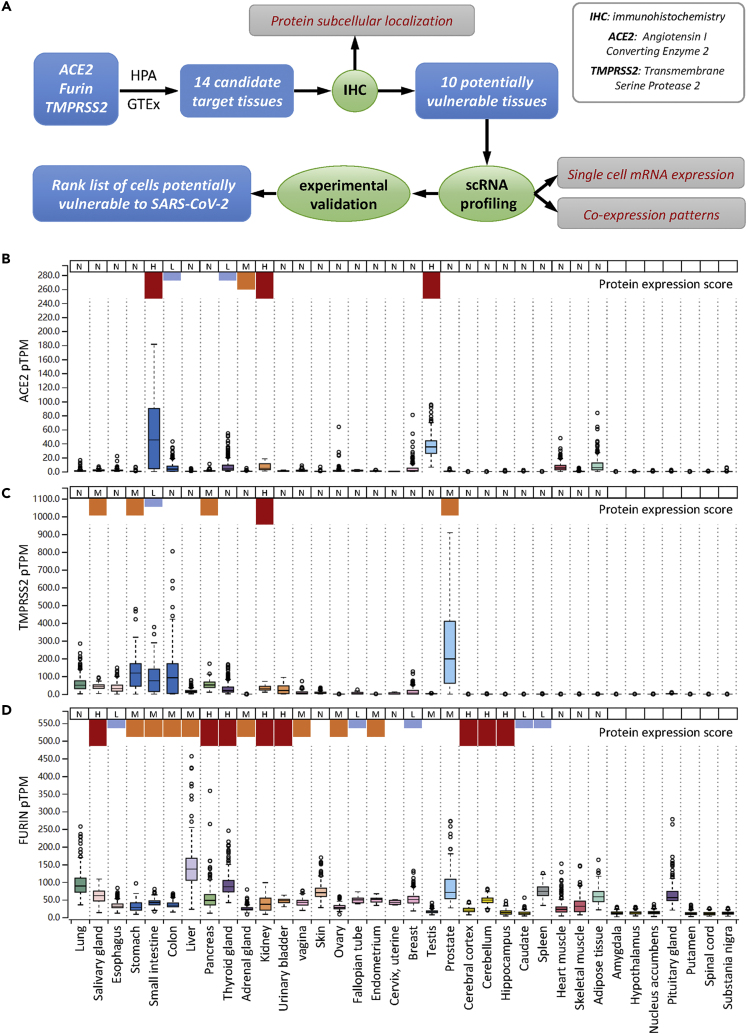
Tissue Distribution of ACE2, TMPRSS2, and Furin Proteases (A) Overview of the analysis framework. (B–D) The mRNA expression level and protein expression score of ACE2 (B), TMPRSS2 (C), and Furin (D) in the manifold tissues and organs. N: negative; L: low expression shown in short blue column; M: median expression, shown in medium-sized orange column; H: high expression, shown in long red column. ACE2: angiotensin I-converting enzyme 2; TMPRSS2: transmembrane serine protease 2; HPA: The Human Protein Atlas; GTEx: The Genotype-Tissue Expression dataset; IHC: immunohistochemistry; scRNA: single-cell RNA; TPM: transcripts per million; pTPM: all TPM values per sample scaled to a sum of 1 million TPM. Note: the RNA expression data were retrieved from GTEx database, the protein expression scores were retrieved from HPA database, in which the protein scores of last seven tissues are missing, therefore not indicated. Figure S1 shows RNA and protein expression on more tissues from HPA database.

As depict in Figure 1A, to achieve a comprehensive analysis of tissue cells potentially vulnerable to SARS-CoV-2 virus, we employed a step-in strategy, i.e., from tissue to cell, from multiple cells to single cell, from protein to mRNA, from single gene expression to co-expression. During analysis, we primarily focused on the expression of ACE2 while taking into account its co-expression with TMPRSS2 and Furin, two proteases that were believed to facilitate SARS-CoV-2 infection. To evaluate the cell vulnerability, not only the mRNA levels but also the protein levels were considered. The protein levels actually take more weights as protein is the main function executor. Moreover, not only protein levels but also their subcellular localizations in a specified type of cell were considered, because the subcellular localization determines the routes whereby viruses might access the protein receptor. For instance, apical localized surface protein would primarily be accessed by viruses from the luminal side, but not from the bloodstream, which is the more likely infection route of unpolarized stromal cells. By following the aforementioned principles, we first examined tissue distribution of ACE2, TMPRSS2, and Furin in both RNA and protein levels and then analyzed their expressions *in situ* by immunohistochemistry (IHC), which could provide information on both protein levels and subcellular localization. Subsequently, single-cell RNA profiling was performed to determine and confirm cell type and co-expression pattern. Finally, a rank list was proposed by integrating information from RNA and protein levels, protein subcellular localizations, cell types and co-expression pattern, as well as the available experimental evidences and clinical manifestations.

Based on the expression analysis across 36 human tissues, ACE2 displayed a tissue-specific expression at both mRNA and protein levels. A total of 10 tissues expressed relatively higher level of ACE2 mRNA, including the esophagus, small intestine, colon, thyroid gland, kidney, ovary, breast, testis, heart muscle, and adipose tissue. Meanwhile, the majority of the other tissues, such as the lung, liver, pancreas, and skin, had the marginal expression of ACE2 mRNA (Figures 1B, S1A, and S1B and Table S1). The protein expression score, although also displaying a tissue-specific pattern, only indicated that six tissues expressed ACE2 protein, with only three of them matching the mRNA expression, including the small intestine, kidney, and testis. Interestingly, whereas the adrenal gland slightly expressed mRNA, it had a median level of ACE2 protein expression. This inconsistency was true for ACE2 to other tissues such as the breast, heart muscle, and adipose tissue, which expressed high levels of ACE2 mRNA but had undetectable levels of ACE2 protein.

The expression of TMPRSS2 also had a tissue-specific pattern, but it was different from that of ACE2 in terms of tissue distribution (Figures 1C, S1A, S1C, and S1F and Table S1). There were six tissues co-expressing relatively high mRNA levels of TMPRSS2 and ACE2, include the esophagus, small intestine, colon, thyroid gland, kidney, and breast. At the protein level, only the small intestine and kidney showed co-expression. Interestingly, the prostate expressed the highest level of TMPRSS2 mRNA but had an undetectable level of ACE2 protein. These results suggest that TMPRSS2 and ACE2 are not often co-expressed in the same tissues. Notably, TMPRSS2 mRNA was considerably expressed in the lung, the target tissue of SARS-CoV-2 virus, which was consistent with a promoting role of TMPRSS2 in SARS-CoV-2 infection.

When compared with ACE2 and TMPRSS2, the expression of Furin protease was much less specific at both the mRNA and protein levels, although some tissues, such as the liver and lung, did express much higher than the others. Regarding the six tissues co-expressing TMPRSS2 and ACE2, the esophagus, small intestine, colon, thyroid gland, and kidney appeared to simultaneously express high levels of Furin mRNA or protein (Figures 1D, S1A, S1D, and S1G and Table S1).

### Expression of ACE2, TMPRSS2, and Furin *In Situ* in Tissues

**Figure 2:**
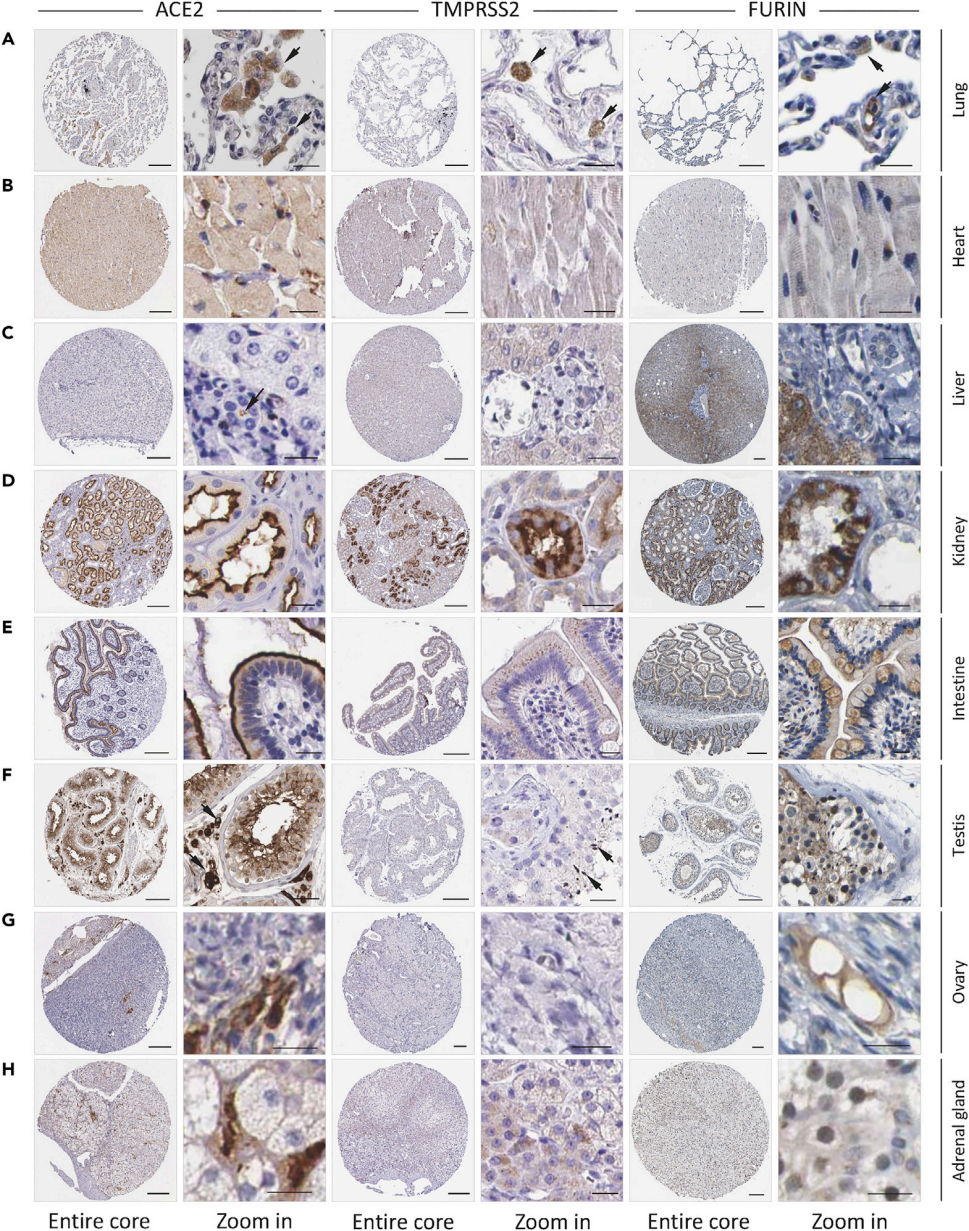
Protein Expression of ACE2, TMPRSS2, and Furin *In Situ* in Tissues (A–H) The IHC images for the protein expression of ACE2, TMPRSS2, and Furin in the indicated tissues/organs. F. adrenal gland: fetal adrenal gland. Scale bars: 200 μm for core images, 20 μm for zoom-in images. Arrows indicate the positive IHC signals (note: not all signals were indicated). Please find in supplementary figures image replicates (Figure S2) and images of more tissues (Figure S3).

Tissues generally comprise multiple types of cells; therefore, the expression level of a specified gene at the tissue scale may not be representative of its level at a certain type of cell. To address this issue, we first analyzed the protein expression of ACE2, TMPRSS2, and Furin *in situ* from IHC images. On the basis of the analysis results above, tissues highly or moderately expressing ACE2 either at the mRNA or protein level, including lung, esophagus, small intestine, colon, thyroid gland, adrenal gland, kidney, ovary, breast, testis, heart muscle, and adipose tissue, were incorporated for further analysis. In addition, the putative SARS-CoV-2 target tissues proposed by previous studies like the liver, irrespective of ACE2 expression level, were also included. Considering the continuity and similarity of tissues of digestive tract such as esophagus, stomach, and intestine, the gastric tissue was also included despite the relatively low level of ACE2 RNA expression. These resulted in a list of 14 tissues in total (Figures 2 and S2–S6), and quantitative analysis was performed by mean intensity of positive area to compare the relative protein expression level of IHC images (Figure S7).

Based on the mean intensity of ACE2, these selected tissues could be roughly categorized into three groups, which basically was consistent with the classification based on IHC intensity in the Human Protein Atlas (HPA) database. First, the ACE2-high group, whose mean intensity of ACE2 was more than 200, contained the kidney, small intestine, testis, ovary, colon, and adrenal gland (Figures 2D–2H, S3, S4, and S5C). In this group of tissues, ACE2 was generally expressed at the apical region in certain tissues like the kidney, intestine and colon, facing the luminal surface of epithelial cells (Figures 2D, 2E, S3A-S3B, and S5C), which suggested that efficient host entry of virus could only take place from the luminal side. This rule may not be applied to the testis, ovary, and adrenal gland due to the high level of ACE2 expression mainly in the stromal cells (Figures 2F–2H, S3C and S4), which are not polarized and could be potentially accessed by virus only from the bloodstream. For another, the ACE2-median group, whose mean intensity of ACE2 was between 100 and 200, contained the lung, heart, stomach, and thyroid gland. For the lung, the major target of SARS-CoV-2 virus, there were considerable amounts of ACE2-positive cells in the alveolus lumen, which morphologically resembled macrophages in most cases (Figures 2A and S2A). All the cardiomyocytes and a portion of stromal cells in the thyroid gland significantly expressed ACE2 (Figures 2B, S2B, and S6C), implicating the potential virus target in the presence of viremia. Furthermore, the luminal surface of epithelial cells in gastric tissues also moderately expressed the ACE2 protein, which suggested the potential vulnerability to SARS-CoV-2 from the luminal side (Figure S5B). Finally, the ACE2-low group, whose mean intensity of ACE2 was less than 100, contained liver, esophagus, and breast and adipose tissues, which are unlikely to be the direct targets of SARS-CoV-2 virus even though some of them showed high levels of mRNA expression, such as breast (Figure S6A) and adipose tissues (Figure S6B). There was a small number of ACE2-positive cells in the interlobular region of liver tissue (Figures 2C and S2C), probably the cholangiocytes as suggested below in scRNA analysis (Figure 4C).

The expression of TMPRSS2 and Furin generally was also high in certain ACE2-high group tissues such as kidney and intestine (Figures 2D–2E and S3A–S3B). However, TMPRSS2 protein seemed to be expressed at relatively low level in the spermatids of the testis as well as stromal cells of ovary and adrenal gland (Figures 2F–2H, S3C, and S4), suggesting different probabilities for them to establish efficient infection by SARS-CoV-2 virus during viremia. For the ACE2-median group, whereas Furin was readily detected in all the tissue cells, TMPRSS2 expression could be readily detected in lung macrophages (Figures 2A and S2A), cardiomyocytes (Figures 2B and S2B), and glandular epithelial cell of the stomach (Figure S5B). For the ACE2-low group, TMPRSS2 and Furin were also expressed at low levels (Figures S6A and S6B) with the exception of the liver, where the hepatocytes expressed Furin at quite high level (Figure 2C).

Together, based on the above analysis, we propose that lung macrophages, in addition to the well-known AT2 cells, may be another direct target of SARS-CoV-2 virus. In the presence of viremia, the top vulnerable targets might be the heart and adrenal gland, and then are the less likely testis, ovary, and thyroid gland. Other tissues are either unlikely direct targets or incompetent for establishing efficient infection due to lack of access to virus or low expression of helping proteases.

### Identification of Cell Types in Tissues and Organs by scRNA Expression Profiling

To obtain comprehensive analysis of the target cells of SARS-CoV-2, we utilized the curated public databases to perform scRNA profiling of the ACE2-high and ACE2-medium tissues, including the lung, heart, kidney, intestinal tract (ileum, rectum, colon), stomach, testis, ovary, and thyroid and adrenal glands. In addition, we also included the liver, the ACE2-low organ, in scRNA analysis given its important clinical implications. After quality filtering (see Transparent Methods), we obtained a total of 147,726 cells and annotated 79 cell types involving the respiratory, circulatory, digestive, urinary, reproductive, and endocrine systems.

**Figure 3:**
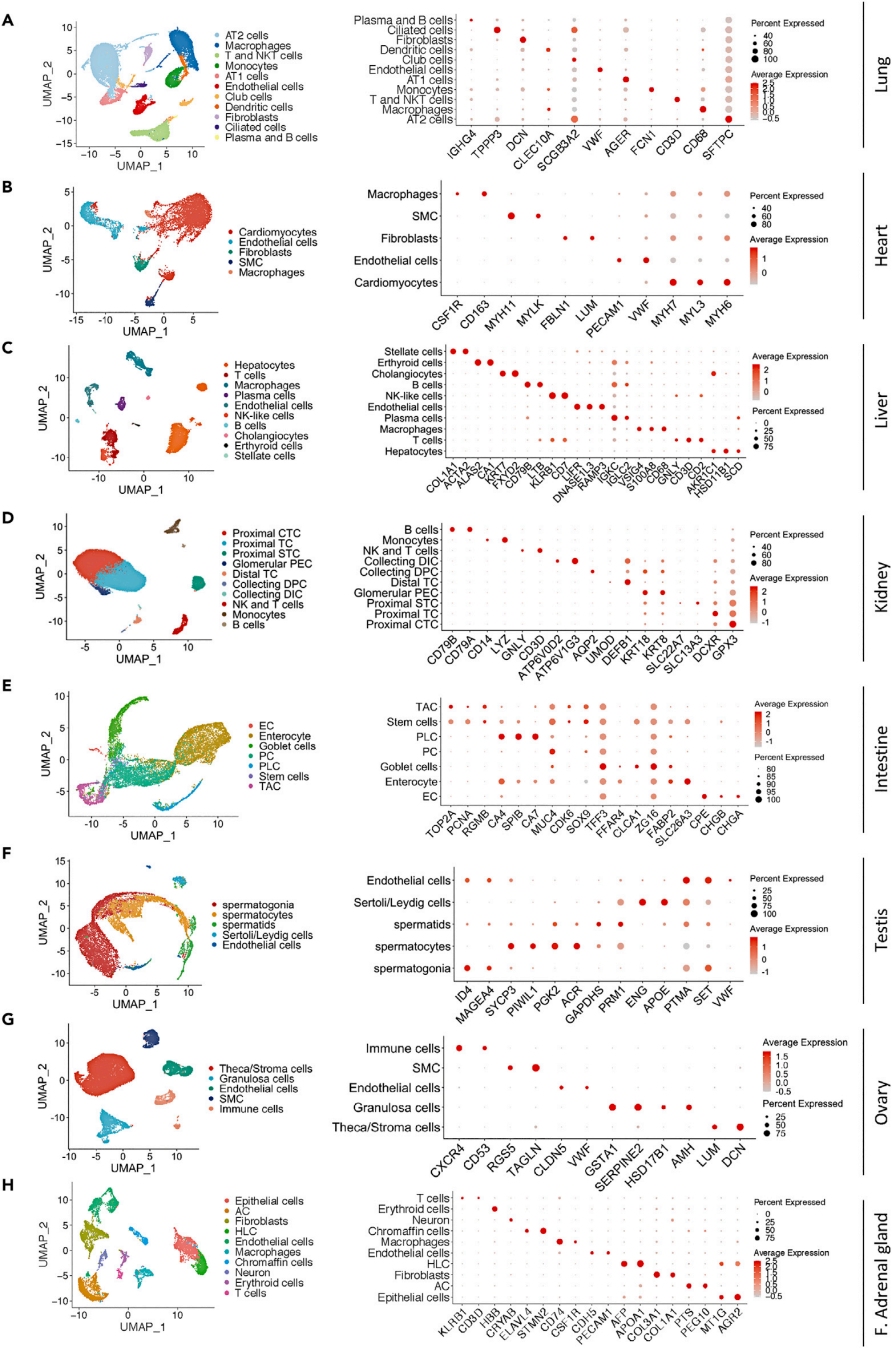
Single-Cell Profiling of the Cell Types in Tissues and Organs (A–H) UMAP plots on the left panel show single-cell transcriptomic profiling of the cell types from the lung (A), heart (B), liver (C), kidney (D), intestine (E), testis (F), ovary (G) and fetal adrenal gland (H). Dot plots on the right panel represent the expression of canonical marker genes of each cell type in the indicated tissues/organs. AT2: alveolar type II, AT1: alveolar type I, SMC: smooth muscle cells, NK: natural killer, CTC: convoluted tubule cells, TC: tubule cells, STC: straight tubule cells, PEC: parietal epithelial cells, TC: tubule cells, DPC: duct principal cells, DIC: distal tubule cells, EC: enteroendocrine cells, PC: Paneth cells, PLC: Paneth-like cells, TAC: transient amplifying cells, AC: adrenocortical cell, HLC: hepatocyte-like cell, F. adrenal gland: fetal adrenal gland.

The lung, as the pivotal respiratory organ, is one of the target organs of SARS-CoV-2. In the lung dataset, 28,819 cells from three donors passed stringent quality control and represented 11 cell types. Specifically, we identified alveolar type II (AT2) cells, alveolar type I (AT1) cells, ciliated and club cells, alveolar macrophages, dendritic cells, monocytes, fibroblasts, endothelial cells, T and NKT cells, and plasma and B cells. Expression of canonical cell markers for pulmonary cell types was observed in largely non-overlapping cells, including SFTPC for AT2 cells, AGER for AT1 cells, TPPP3 for ciliated cells, SCGB3A2 for club cells, CD68 for macrophages, VWF for endothelial cells, CD3D for T and NKT cells, and IGHG4 for plasma and B cells (Figure 3A). In the heart dataset, we obtained 8,148 high-quality cells consisting of 5 prime cell types, including cardiomyocytes, endothelial cells, fibroblasts, smooth muscle cells (SMC), and macrophages on the basis of their respective molecular features (Figure 3B).

As such, the cell population of hepatic tissues from 5 human livers was classified into 10 cell types based on the expression of known markers, which was shown in Figure 3C. In addition, 23,366 kidney cells from 3 donors were identified and grouped into 10 cell types. The detailed cell categories of kidney and the expression of specific cell markers were shown in Figure 3D.

For integrative analysis of the human intestinal mucosa at single-cell level, we pooled 14,207 intestinal cells together (6,167 cells from two ileum samples, 4,411 cells from two colon samples, and 3,629 cells from two rectum samples). In total, these cells were partitioned into 7 main cell types, containing enteroendocrine cells (EC), enterocytes, goblet cells, Paneth cells (PC), Paneth-like cells (PLC), stem cells, and transient amplifying cells (TAC) (Figure 3E). Additionally, we also investigated the gastric mucosa dataset incorporating 5,281 cells and finally annotated 9 cell types (Figures S8A and S8B).

To characterize the single-cell profiling of human reproductive organs, the testis and ovarian datasets were thoroughly analyzed. In the testis datasets from adult human sorted spermatogonia, spermatocytes, and spermatids, we identified 12,829 cells, which were classified into 5 major cell populations for downstream analysis (Figure 3F). Similarly, we exploited 27,857 cells from 5 adult women ovaries and defined 5 major cell types, including theca cells, granulosa cells, endothelial cells, SMC, and immune cells (Figure 3G).

The adrenal gland and thyroid, as the significant parts of the endocrine system, were also included into our research. We took advantage of 9,809 cells derived from 10 cell types in the fetal adrenal gland and 8,966 cells from 7 main cell types in the thyroid to construct the single-cell atlas (Figures 3H and S8H). Based on the expression of specific cell markers, we found that the atlas of the fetal adrenal gland mainly comprised epithelial cells, adrenocortical cells (AC), hepatocyte-like cells (HLC), endothelial cells, macrophages, chromaffin cells, neuron, erythroid cells, and T cells, whereas the atlas of the thyroid mainly consisted of thyroid follicular cells, endothelial cells, T cells, endothelial cells, SMC, dendritic cells, fibroblasts, and neutrophils (Figures 3H and S8H). In addition, it was shown that these cell types were annotated by their specific cell markers (Figures 3H and S8I).

Overall, we established the cell atlases of 10 organs or tissues that were the potential targets of SARS-CoV-2, which provides a valuable approach to unravel the vulnerable cell types to this virus.

### Single-Cell Transcriptomic Profiling of ACE2, TMPRSS2, and Furin Proteases in Distinct Cell Types

**Figure 4:**
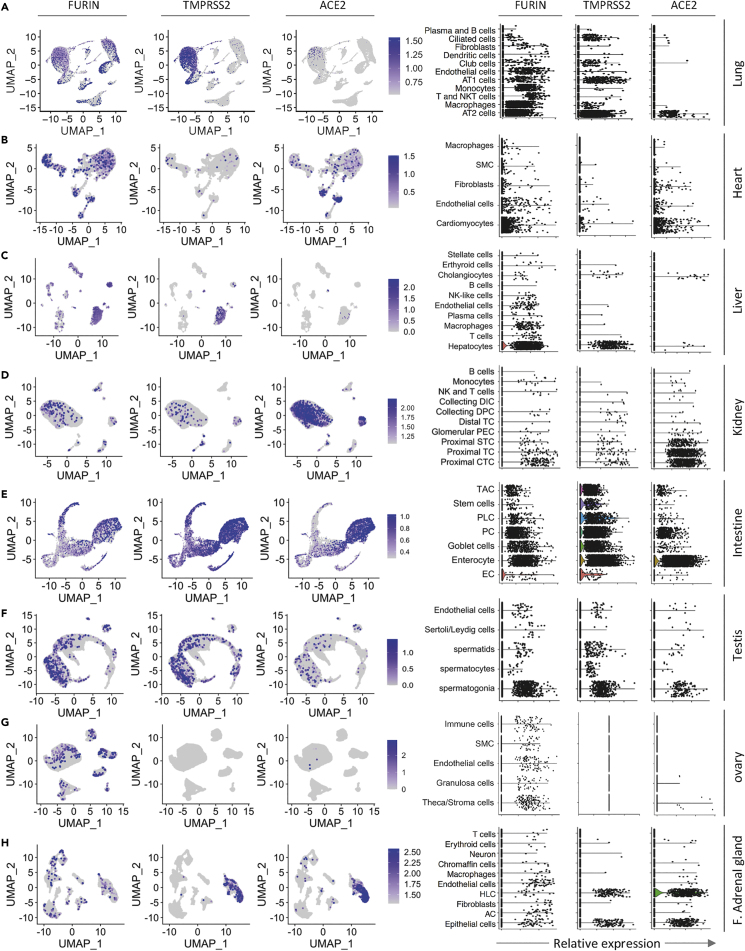
Single-Cell Transcriptomic Profiling of ACE2, TMPRSS2, and Furin Proteases in Distinct Cell Types (A–H) UMAP plots (left panel) show the mRNA expression of ACE2, TMPRSS2, and Furin genes as displayed on the top of each column of graphs in different cell clusters from the lung (A), heart (B), liver (C), kidney (D), intestine (E), testis (F), ovary (G) and fetal adrenal gland (H). Violin plots (right panel) illustrate the gene expression of Furin, TMPRSS2, and ACE2 as shown on the top of each column of graphs in different cell types in the indicated tissues or organs. AT2: alveolar type II, AT1: alveolar type I, SMC: smooth muscle cells, NK: natural killer, CTC: convoluted tubule cells, TC: tubule cells, STC: straight tubule cells, PEC: parietal epithelial cells, TC: tubule cells, DPC: duct principal cells, DIC: distal tubule cells, EC: enteroendocrine cells, PC: Paneth cells, PLC: Paneth-like cells, TAC: transient amplifying cells, HLC: hepatocyte-like cell, AC: adrenocortical cell, F. adrenal gland: fetal adrenal gland.

To determine the potentially vulnerable cell types to SARS-CoV-2 infection, we systematically explored mRNA expression level of ACE2, TMPRSS2, and Furin genes in distinct tissue cells of the established cell atlases above. We found that the expression level of ACE2, TMPRSS2, and Furin genes varied significantly across the tissues and organs analyzed (Figure 4). Importantly, in certain tissues, these genes were only expressed in particular cell types. For example, the SARS-CoV-2 receptor ACE2 was mainly expressed in AT2 cells in the lung (Figure 4A), cardiomyocytes in the heart (Figure 4B), and cholangiocytes and hepatocytes in the human liver (Figure 4C), which was consistent with results of previous studies (Chai et al., 2020; Zhao et al., 2020) and confirmed our above analysis on the protein expression of ACE2. We further investigated the mRNA expression of TMPRSS2 and Furin proteases and confirmed that these genes were also expressed in the liver cholangiocytes, cardiomyocytes, and AT2 cells (Figures 4A, 4B, and 4C). Moreover, we observed that the expression level of the ACE2 gene in the kidney was high, especially in certain cell types including proximal convoluted tubule cells (CTC), proximal tubule cells (TC), and proximal straight tubule cells (STC) (Figure 4D). Quantitatively, we found that 5.93% (1,201/20,238) of all proximal CTC, proximal TC, and proximal STC expressed ACE2 gene. However, only 0.51% (103/20,238) and 0.92% (187/20,238) of these cells, respectively, expressed TMPRSS2 and Furin genes in the kidney (Figure 4D), which is inconsistent with the IHC results (Figure 2D). Furthermore, in intestine, ACE2 was mainly expressed in 48.44% (2,770/5,719) of enterocytes and 12.78% (531/4156) of PC. Meanwhile, TMPRSS2 and FURIN genes were highly expressed in enterocytes and PC (Figure 4E). It was suggested that enterocytes and PC are more likely to become target cells of SARS-CoV-2 than the proximal CTC, proximal TC, and proximal STC in the kidney.

Notably, the spermatogonia moderately expressed the ACE2 gene (1.95%) as well as Furin (6.49%) and TMPRSS2 (5.80%) (Figure 4F), whereas only 1.78% (7/394) of the Sertoli/Leydig cells expressed ACE2, which was consistent with IHC results indicated above. By contrast, we found that only stroma cells expressed the ACE2 and Furin genes in the adult ovary (Figure 4G). Moreover, the significant expression of the ACE2 gene was also shown in the cells of the fetal adrenal gland, specifically in HLC and epithelial cells, as well as the significant expression of the TMPRSS2 and Furin genes (Figure 4H). In contrast, in the thyroid, there was little expression of the ACE2 despite the high expression of TMPRSS2 and Furin (Figures S8J and S8K), which disagreed with the IHC results that ACE2 was highly expressed in stromal cells of thyroid gland (Figure S6C). These results show that the fetal adrenal gland is more likely vulnerable to the SARS-CoV-2 virus via epithelial cells and HLC cells.

Taken together, we comprehensively analyzed the expression of ACE2, TMPRSS2, and Furin genes in different tissue cells by feat of the scRNA-seq profiling and laid the foundation for identifying the target cells of SARS-CoV-2.

### Characterization of the Co-expression Features of ACE2, TMPRSS2, and Furin

Considering the fact that cell receptors like ACE2, together with the proteases such as TMPRSS2 and Furin, are required for the SARS-CoV-2 virus to efficiently infect cells, it is imperative to characterize the co-expression patterns between ACE2, TMPRSS2, and Furin. Accordingly, we resorted to single-cell transcriptomes to profile their co-expression patterns in the cell types indicated.

**Figure 5:**
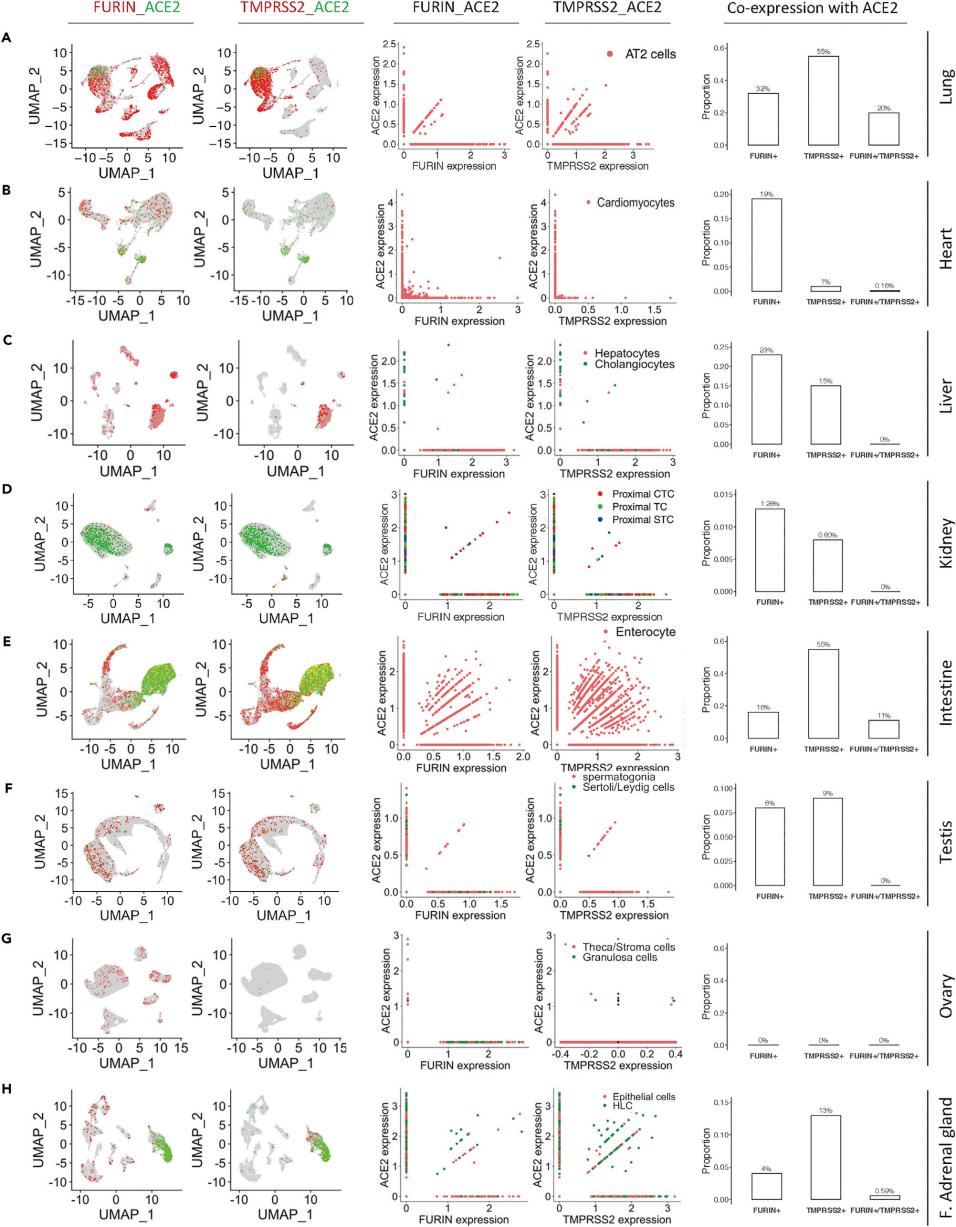
Characterization of the Co-expression Patterns of ACE2, TMPRSS2, and Furin at Single-Cell Level (A-H) UMAP plots (left panels) show the co-expression of ACE2 with Furin or TMPRSS2 in the indicated tissues/organs. The different colors in UMAP plots are corresponding to different genes as indicated on the top of each graph column. Scatterplots (two middle panels) illustrate the expression correlation of ACE2 with Furin or TMPRSS2 in the AT2 cells (A), cardiac cardiomyocytes (B), liver hepatocytes and cholangiocytes (C), the proximal tubule cells of kidney (D), the enterocyte of intestinal tract (E), the spermatogonia and Sertoli/Leydig cells in the testis (F), the stroma cells and granulosa cells in the ovary (G), and the epithelial cell and HLC in the fetal adrenal gland (H). The colors in scatterplots indicate the corresponding cell types depicted on the upper right corner. Barplots (right panels) show the proportion of ACE2-positive cells expressing either or both FURIN and TMPRSS2. The number above the bar in the barplots indicates the corresponding percentage. CTC: convoluted tubule cells, TC: tubule cells, STC: straight tubule cells, HLC: hepatocyte-like cell, F. adrenal gland: fetal adrenal gland.

In the ACE2-medium tissues, we found that the lung displayed the significant co-expression features, especially in AT2 cells, and the highest percentage of ACE2-positive cells in the lung expressed either or both TMPRSS2 and Furin in the investigated organs and tissues (Figure 5A), supporting the lung tissue as the top rank of tissues potentially vulnerable to SARS-CoV-2. Furthermore, consistent with the results above, a portion of cardiomyocytes simultaneously expressed ACE2 and Furin (19%, Figure 5B), ranking the cardiomyocytes a position after the AT2 cells. The ACE2-low liver tissue displayed an interesting expression pattern, showing that the co-expression feature of ACE2 with Furin and TMPRSS2 was 23% and 15%, respectively (Figure 5C), but the basal expression level with ACE2 in both RNA and protein level was rather low (Figures 1B and 4C), arguing against the liver as a primary SARS-CoV-2 target.

Furthermore, it was showed that the co-expression feature of these three genes were quite dominant in the intestine, specifically in enterocytes (Figure 5E), and the proportion of ACE2-positive cells expressing either or both TMPRSS2 and Furin in the intestine is higher than the other ACE2-high tissues such as the kidney (Figure 5D), testis (Figure 5F), and stomach (Figures S8E–S8G). In detail, as shown in Figure 5D, although the cells in the kidney expressed ACE2, TMPRSS2, and Furin at moderate to high levels, there was only a small proportion of ACE2-positive cells expressing either or both TMPRSS2 and Furin, arguing against their vulnerability to SARS-CoV-2. Similarly, few cells co-expressed ACE2, TMPRSS2, and Furin simultaneously in the ovary (Figure 5G) and thyroid gland (Figures S8M–S8N), suggesting that these tissues are in a lower position in the rank list. Moreover, we observed a significant co-expression of the ACE2 gene with TMPRSS2 and Furin in the epithelial cells and HLC of the fetal adrenal gland (Figure 5H), manifesting that the fetal adrenal gland may be a target of SARS-CoV-2. Therefore, we rank the adrenal gland in the middle among the target tissues of SARS-CoV-2.

Overall, the characterization of co-expression features of ACE2, TMPRSS2, and FURIN provides the important information for the rank list of tissue cells potentially vulnerable to SARS-CoV-2 infection.

### The Stomach Is a Barrier against SARS-CoV-2 Virus

**Figure 6:**
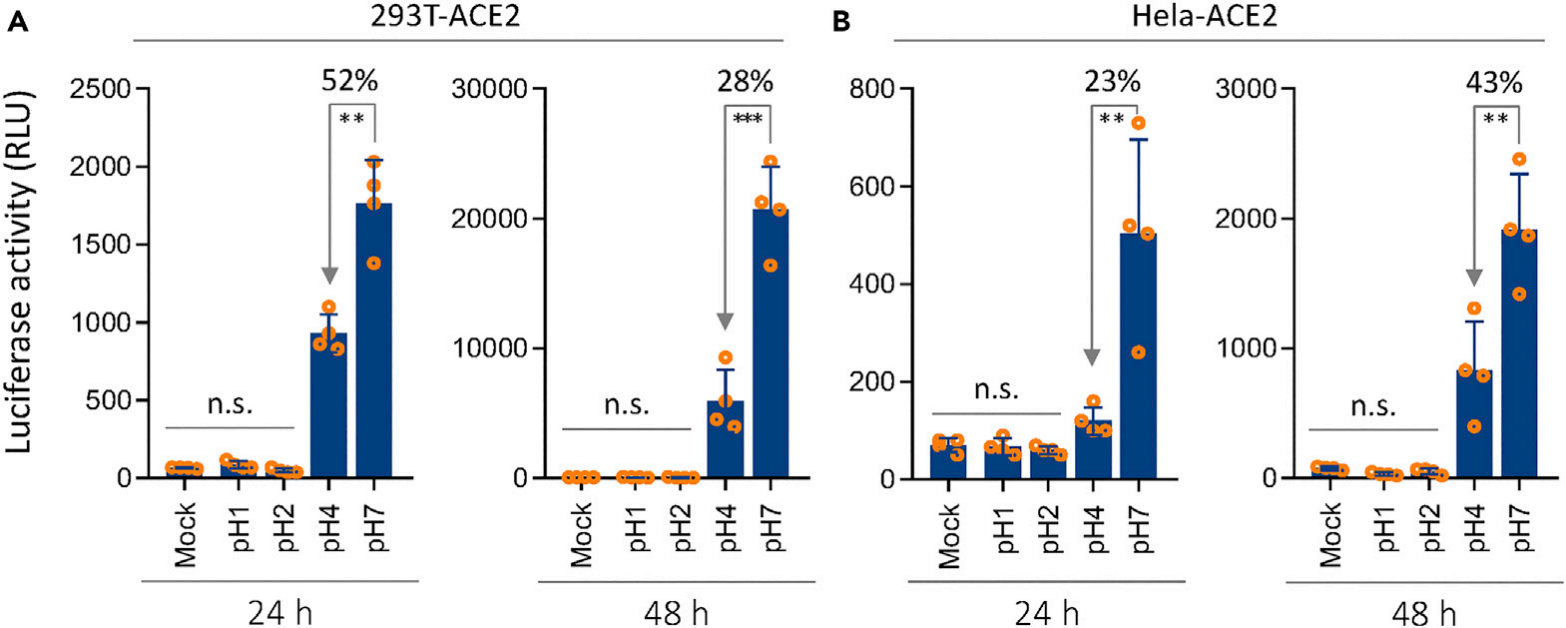
Compromised Infectivity of SARS-CoV-2 Pseudo-virus in Acidic Environment (A and B) The effects of low pH on the activities of SARS-CoV-2 pseudo-virus to infect 293T-ACE2 cells (A), or Hela-ACE2 cells (B), as determined by luciferase assay 24 h (left) and 48 h (right) postinfection. n.s.: not significant; ∗∗p < 0.01; ∗∗∗p < 0.001.

Although the human enterocytes express high levels of ACE2, TMPRSS2, and FURIN at both RNA and protein levels, they may not be a primary target of SARS-CoV-2. As we know that the normal stomach is highly acidic, it may conceivably serve as a physical barrier against SARS-Co-V-2 virus. To test this idea, we made viruses pseudotyped with SARS-CoV-2 spike protein. The same amounts of pseudo-viruses were pretreated under acidic conditions with pH of 1.0, 2.0, 4.0, or 7.0, respectively, and then used to infect 293T-ACE2 cells and Hela-ACE2 cells. As shown in Figure 6, the viruses were completely inactivated and incapable of infecting both types of cells anymore under the pH of 1.0 and 2.0, a condition resembling the normal acidity of fasting stomach (Weiss et al., 1985). Even under the pH of 4.0, the viral infectivity was significantly compromised to 23%–52% of the normal level. Thus, the SARS-CoV-2 virus was likely acid-instable, which is consistent with clinically uncommon symptom in gastrointestinal tract.

## Discussion

Previous analysis based solely on the mRNA expression of ACE2 suggested that multiple tissue cells may be potential targets of the novel coronavirus SARS-CoV-2, including cells from almost every important human system. However, the COVID-19 patients primarily display symptoms in the respiratory system, where ACE2 is expressed in only a small portion of cells. Even for severely ill patients, the injuries or symptoms in the ACE2-high organs such as the kidney and intestinal tract are relatively uncommon, with a rate of 1%–4.3% for acute kidney injury (Guan et al., 2020; Wang et al., 2020) and a rate of 3.5%–5.8% for intestinal symptoms (Guan et al., 2020; Special Expert Group for Control of the Epidemic of Novel Coronavirus Pneumonia of the Chinese Preventive Medicine, 2020). Instead, the injuries in ACE2-median organs such as the heart are more common (12%–19%) (Guan et al., 2020; Special Expert Group for Control of the Epidemic of Novel Coronavirus Pneumonia of the Chinese Preventive Medicine, 2020). This obvious discrepancy suggests that mechanisms other than ACE2 expression level also play important roles in establishing efficient and successful infection. Based on our analysis by pscRNA expression profiling, we propose that three more factors, in addition to the mRNA level, should be taken into account for predicting the vulnerability of a specified target tissue/organ to SARS-CoV2 infection.

First, the information on the protein expression level and protein subcellular localization may influence virus infection. As shown above, the mRNA level did not often correspond to protein level, for example, the ACE2 expression in lung macrophages (Figures 2A and 4A). Although ACE2 is readily detected in macrophages in IHC staining (Figure 2A), there are only several macrophages positive for ACE2 (Figure 4A). This obvious discrepancy may not result from the limited scRNA samples analyzed, because additional lung scRNA analysis gave results similar to that in Figure 4A (data not shown). Therefore, protein-mRNA expression inconsistency might be accountable, which actually necessitates the pscRNA profiling analysis that incorporates information from protein expression, RNA expression, and biological experimentations as well. In light of this point, the final rank list of SARS-CoV-2 vulnerable cells would be a result of combined consideration. Actually, several recent works (Bost et al., 2020; Zhou et al., 2020c) indicated that macrophages are indeed one major target cell of SARS-CoV-2, which is consistent with our analysis. Therefore, it is necessary to not only analyze mRNA expression but also detect protein expression *in situ*. Moreover, membrane proteins such as ACE2 could be expressed either all around the surface of non-polarized stromal cells such as Leydig cells in the testis (Figure 2F) or specifically on the apical region of polarized epithelial cells such as the enterocytes in the intestinal tract (Figure 2E). This means much for virus infection as it determines how the viruses may get access to their target cells and enter. For the polarized cells like enterocytes, virus could only successfully make infection from the luminal side, where ACE2 is expressed on the apical surface but not the basolateral surfaces. However, it was reported that coronaviruses, such as MERS-CoV, lost infectivity in highly acidic gastric fluid (Zhou et al., 2017), thus, the likelihood that SARS-CoV-2 gets access to enterocytes via stomach would be low. While for the non-polarized stromal cells, virus may readily come to see them from the bloodstream.

Second, the co-expression of infection co-factors determines the efficiency of successful infection. As for SARS-CoV-2, TMPRSS2 and Furin were demonstrated to be important proteases that cleave the S protein to promote host entry (Hoffmann et al., 2020; Meng et al., 2020; Walls et al., 2020). Therefore, their co-expression with ACE2, the cellular receptor for SARS-CoV-2, may dictate the vulnerability of the target tissues. Actually, we found that some ACE2-high cells, such as stromal cells in the testis and ovary, expressed TMPRSS2 at quite low levels (Figures 2F and 2G), suggesting that they may not be SARS-CoV-2 targets as susceptible as those co-expressing both ACE2 and TMPRSS2/Furin proteases, such as cardiomyocytes in the heart, although cardiomyocytes expressed relatively lower level of ACE2. It is conceivable that cells highly co-expressing ACE2, TMPRSS2, and Furin proteases, such as lung macrophages and stromal cells in adrenal gland, would be readily vulnerable to SARS-CoV-2 attack in the presence of viruses.

Third, the feasible routes whereby viruses gain access to their target cells are also accountable for clinical manifestations. In theory, there are two major routes for virus transmission. (1) Direct entry to the luminal tracks via open entries of the body. The potentially affected tissues/organs include those from the respiratory system, digestive system, and urinary tract, among which the respiratory system is much easier than the other two to be infected, as it is almost a completely open system, whereas the remaining two are gated by multiple means. For example, the stomach, which is a highly acidic, may serve as an effective barrier for SARS-CoV-2 to enter the intestinal track by inactivating them (Figure 6). This may explain the prominent symptoms in the respiratory system but not in the digestive and urinary systems despite high levels of co-expression of ACE2, TMPRSS2, and Furin in epithelial cells lining along the tracts of the latter two systems. (2) Transmission via the bloodstream to the entire body. This may theoretically affect all the internal organs with stromal cells expressing ACE2, with exception for those polarized cells in which the ACE2 protein is only expressed on the apical surface unreachable by the viruses from viremia. The target tissue cells affected by this route may include cardiomyocytes, stromal cells in adrenal gland, Leydig cells in the testis, and stromal cells in the ovary and thyroid gland. Among them, only the former two, but not the latter three, are more likely the true or susceptible targets of SARS-CoV-2 when considering the co-expression of helping proteases with ACE2. It should be noted that the precondition for this route is viremia, which, however to the best of our knowledge, was not clearly documented in COVID-19 patients up to date. Nevertheless, frequent heart injuries in COVID-19 patients (Guan et al., 2020; Zhou et al., 2020a) are consistent with the presence of occasional viremia. Under such circumstances, the adrenal gland may be another vulnerable target of SARS-CoV-2, which, to the best of our knowledge, is identified for the first time in this study.

Intriguingly, a recent work by Ziegler et al. (2020) demonstrated that ACE2 is an interferon-stimulated gene, and the immune response stimulated by SARS-CoV-2 infection resulted in upregulated expression of cytokines such as interferon, which subsequently upregulates ACE2 expression in the neighboring cells promoting virus dissemination. Thus, in addition to the basal ACE2-TMPRSS2 co-expression as analyzed in this study, which mediates the initial viral infection, a positive feedback loop between virus infection and interferon signaling would facilitate viral dissemination by increasing ACE2 expression. This finding was further confirmed by two independent studies by Smith et al. (2020) and Wang and Cheng (2020). This factor should also be taken into account when analyzing tissue injuries and clinical symptoms of COVID-19 patients.

In summary, we propose that the pscRNA profiling is a feasible way for gene expression analysis at both protein and mRNA levels. Through a systemic analysis of 36 human tissues/organs by pscRNA profiling of ACE2, TMPRSS2, and Furin proteases, we propose a rank list of tissue cells potentially vulnerable to SARS-CoV-2 attack. For initial infection, ACE2-expressing cells, with the co-expression of TMPRSS2 and Furin as a plus, in the respiratory tract are the primary targets. These cells include the known lung AT2 cells, and macrophages in this study, and also the nasal epithelial cells as reported recently (WU et al., 2020a). And the likelihood of epithelial cells in digestive and urinary systems as the primary targets is low. During a period of viremia, the top internal organ targets would be cardiomyocytes, and stromal cells in the adrenal gland, as both of these express ACE2, TMPRSS2, and Furin proteases; the descent targets may be Leydig cells in the testis, and stromal cells in the ovary and thyroid gland, as these cells are un-polarized and ACE2-positive, but do not show ideal co-expression of TMPRSS2 or Furin proteases. Notably, the identification of the adrenal gland as a SARS-CoV-2 target may be quite informative for clinical practice if experimentally confirmed, because the COVID-19 disease frequently proceeds a severe or very severe stage in a short period, which suggests systemic conditions occur probably due to deregulated endocrine systems involving the adrenal gland. This issue remains to be validated experimentally and clinically.

### Limitations of the Study

By protein-proofed single-cell profiling method, we systematically analyzed 36 human tissues/organs and revealed a rank list of potential SARS-CoV-2 targets. Meanwhile, there are some limitations to the findings of this study. First, in this study, biological confirmation about the cell types' vulnerability to the virus required to be corroborated experimentally. Given the huge amount of tissues and cell types analyzed in this work, it would be technically difficult to make biological confirmation in one study, particularly due to the restricted availability of the normal human tissue samples, which is actually one of the reasons that we would like to utilize the public database for multiple levels of analysis. Second, the sample numbers in several single-cell datasets are rather limited, which may limit the generality of the related conclusion. Nevertheless, the systemic analysis in this study would provide a reference frame for further confirmation.

### Resource Availability

#### Lead Contact

Further information and requests for resources should be directed to and will be fulfilled by the Lead Contact, Qiang Sun (sunq@bmi.ac.cn).

#### Materials Availability

This study did not generate new materials.

#### Data and Code Availability

Availability of supporting data: GEO: GSE122960, GSE109816, GSE131685, GSE125970, GSE134520, GSE109037, GSE134355 and GSE118127.

## Methods

All methods can be found in the accompanying Transparent Methods supplemental file.

## Supplemental Information

Document S1. Transparent Methods, Figures S1–S8, and Tables S1–S4
