# Supplementary material for: SARS-CoV-2 Targets by the pscRNA Profiling of ACE2, TMPRSS2 and Furin Proteases
Source: iScience. 2020 Oct 28;23(11):101744. doi: 10.1016/j.isci.2020.101744 (PMC7591870; doi:10.1016/j.isci.2020.101744)
Supplement: Document S1. Transparent Methods, Figures S1–S8, and Tables S1–S4 [file mmc1.pdf]

iScience, Volume *23*

Supplemental Information

SARS-CoV-2 Targets by the pscRNA Profiling of ACE2, TMPRSS2 and Furin Proteases

Lulin Zhou, Zubiao Niu, Xiaoyi Jiang, Zhengrong Zhang, You Zheng, Zhongyi Wang, Yichao Zhu, Lihua Gao, Hongyan Huang, Xiaoning Wang, and Qiang Sun

Supplemental figures

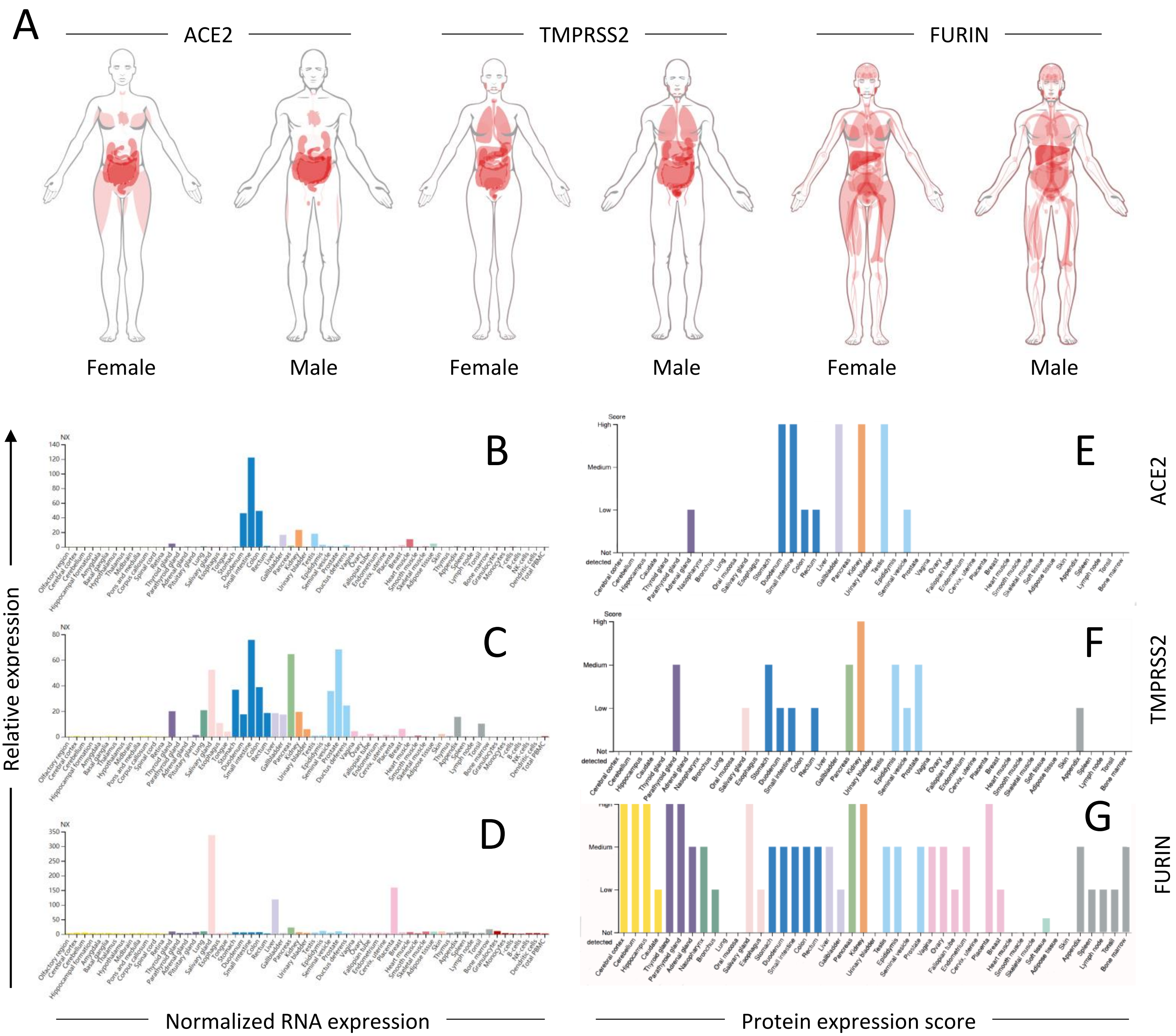

Figure S1

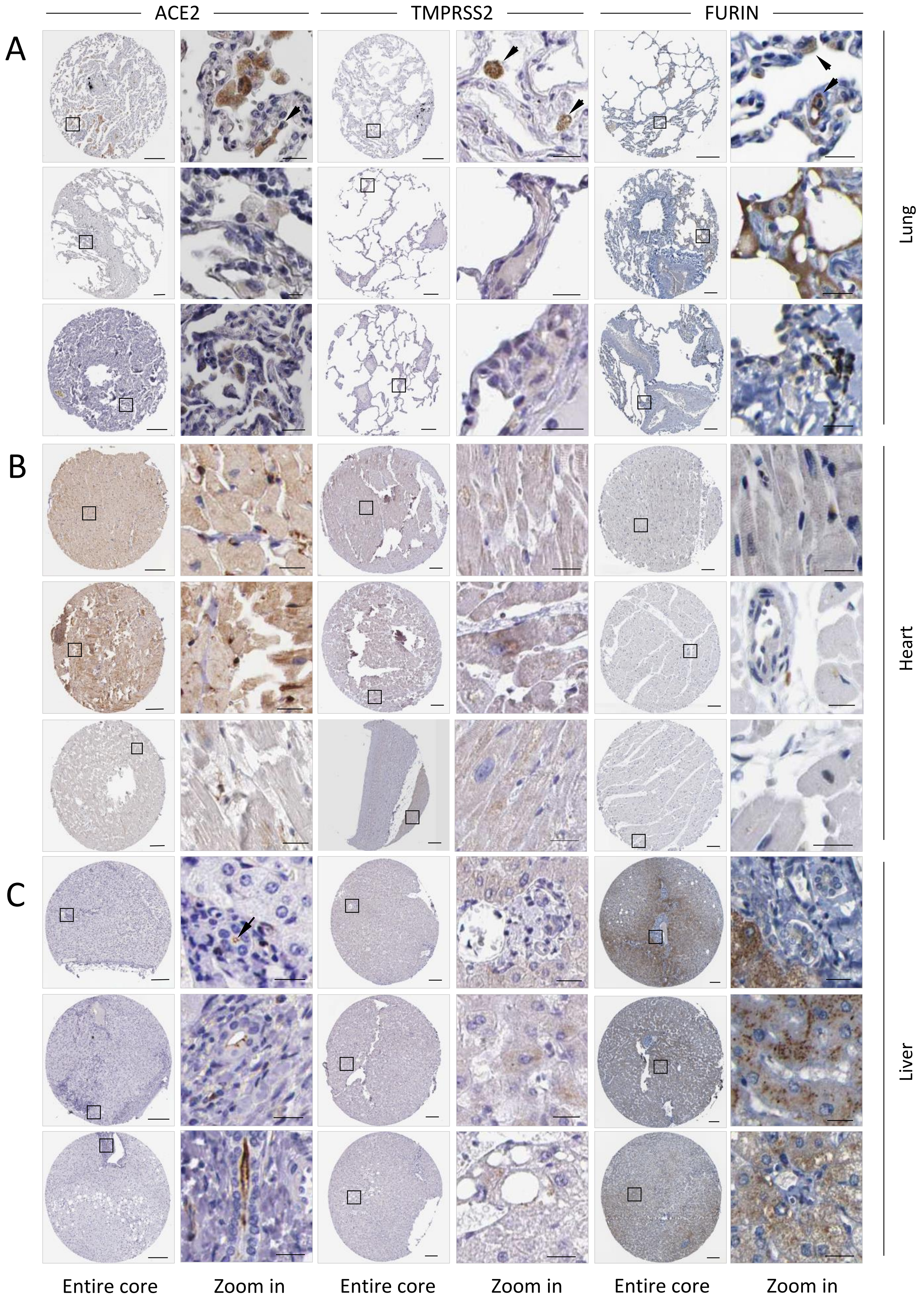

ACE2
TMPRSS2
FURIN
A
B
C
Lung
Heart
Liver
Entire core
Zoom in
Entire core
Zoom in
Entire core
Zoom in

Figure S2

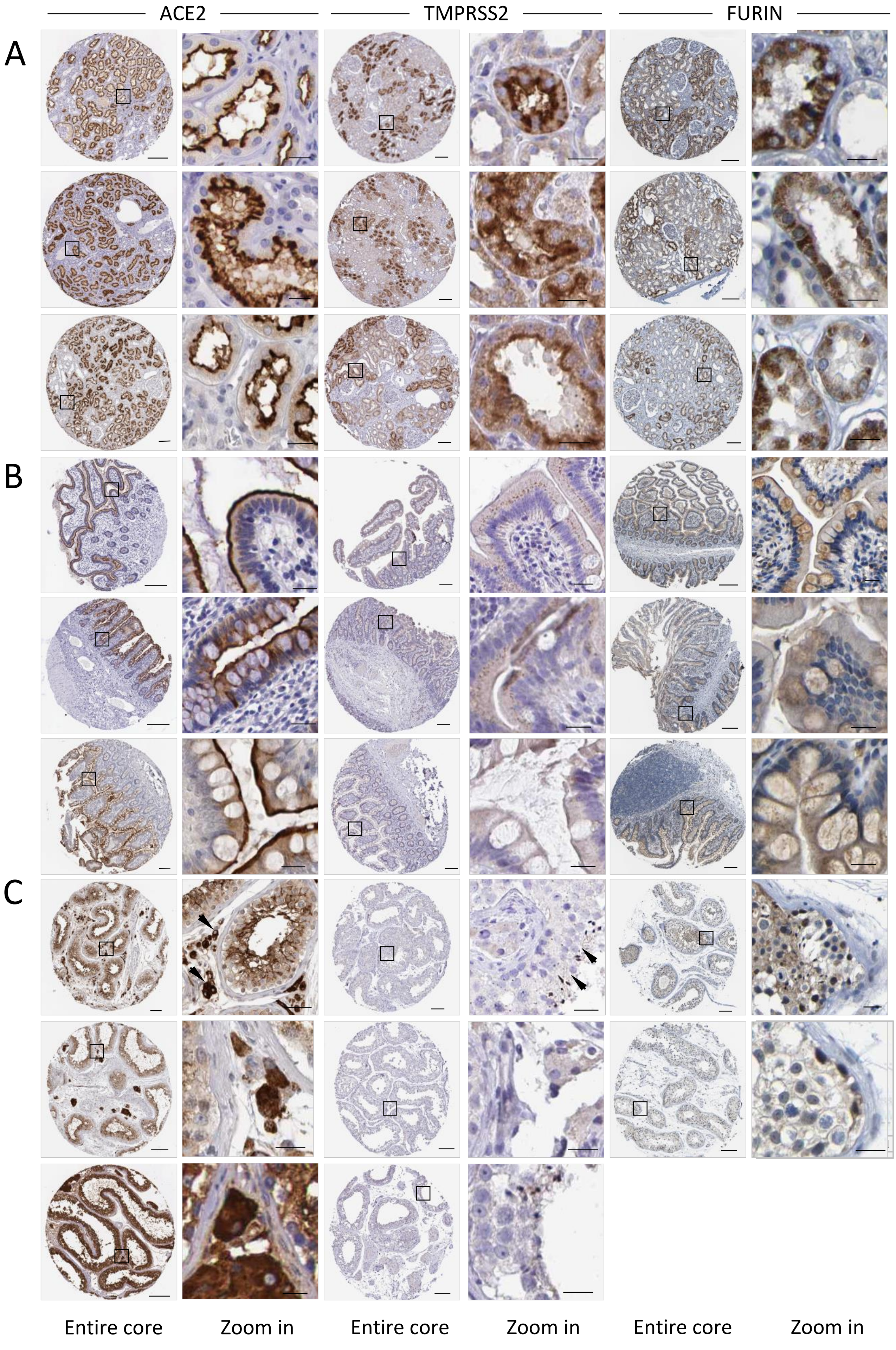

ACE2
TMPRSS2
FURIN
A
B
C
Kidney
Intestine
Testis
Entire core
Zoom in
Entire core
Zoom in
Entire core
Zoom in

Figure S3

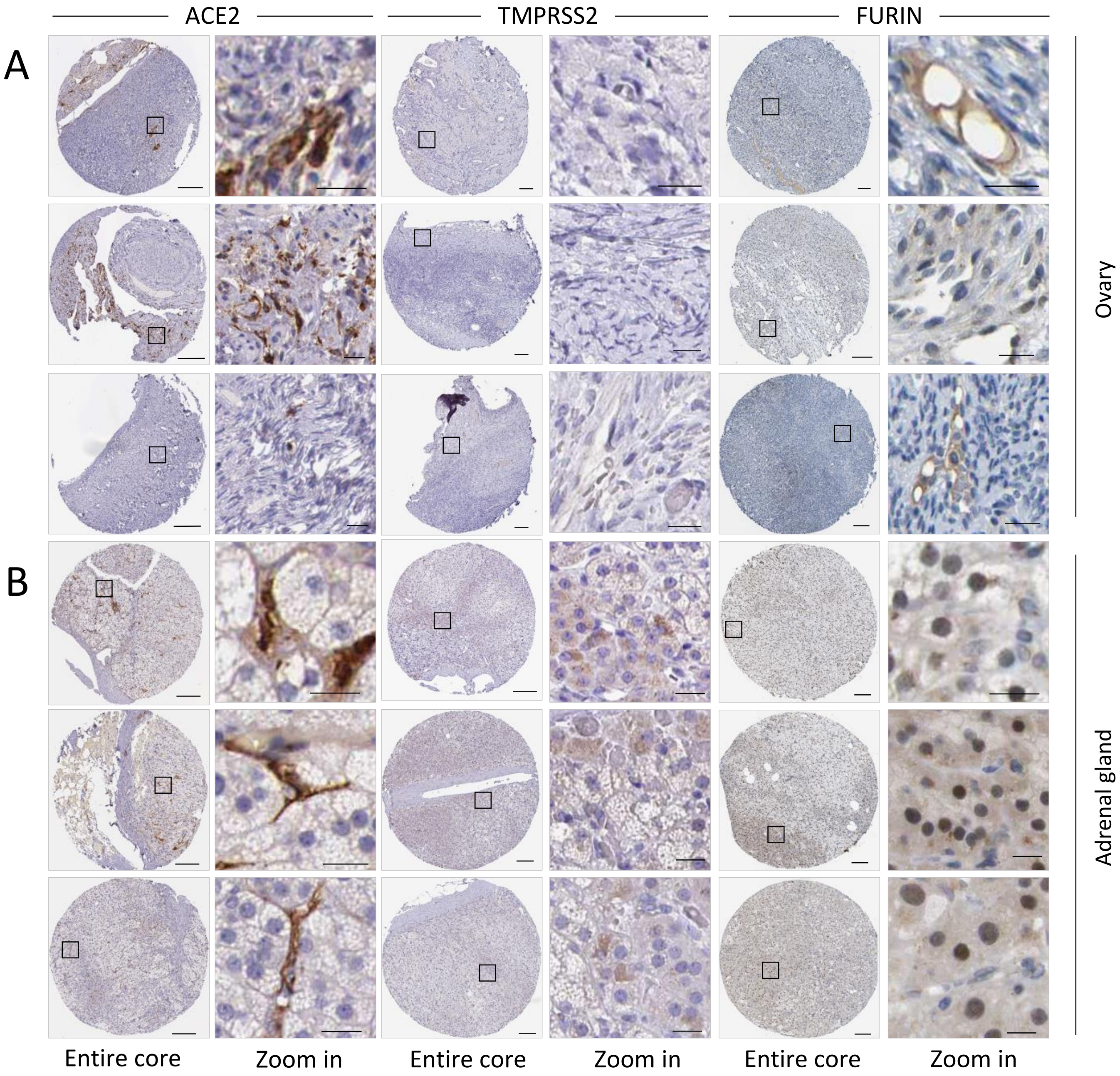
ACE2
TMPRSS2
FURIN
A
B
Ovary
Adrenal gland
Entire core
Zoom in
Entire core
Zoom in
Entire core
Zoom in

Figure S4

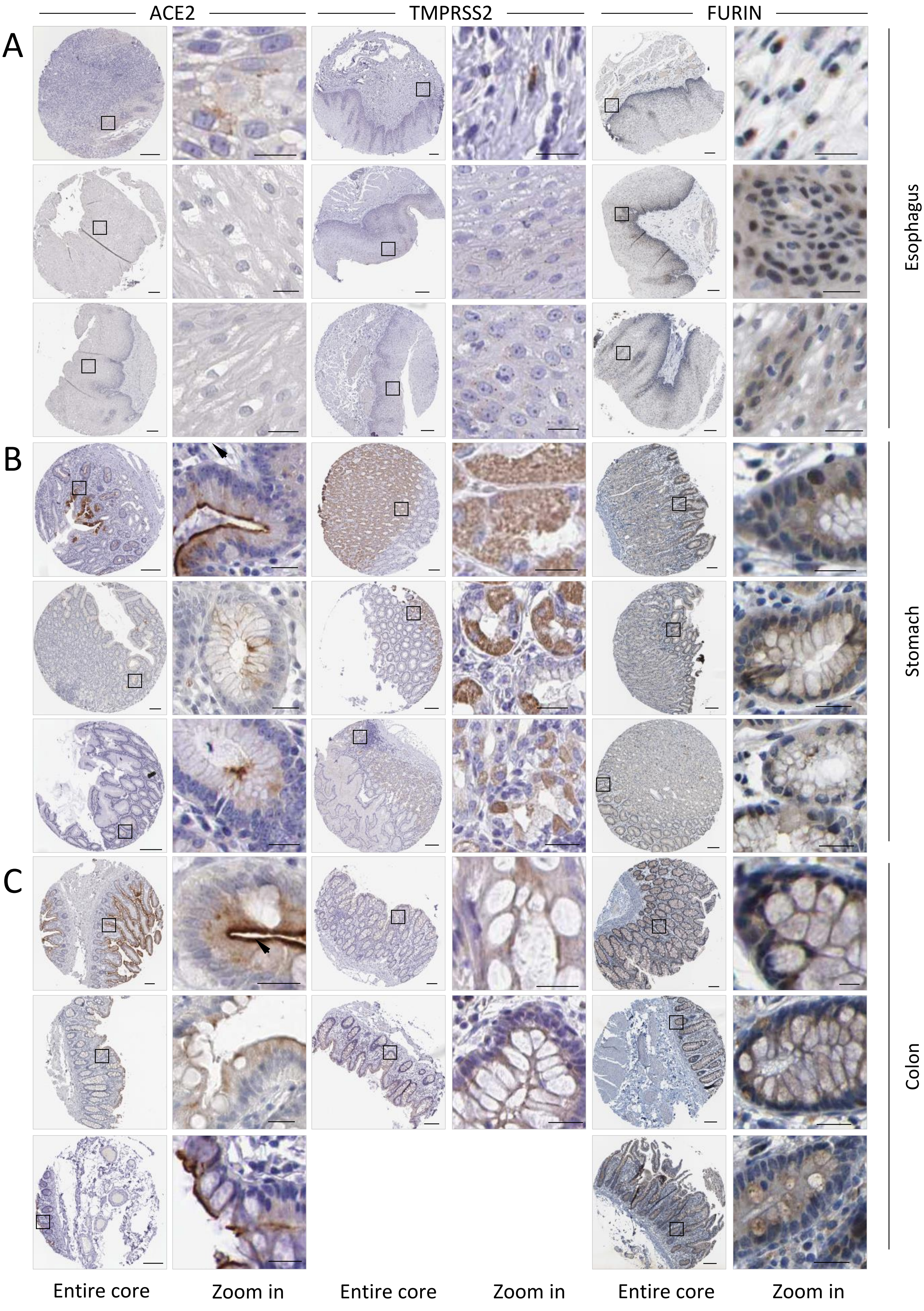
ACE2
TMPRSS2
FURIN
A
B
C
Esophagus
Stomach
Colon
Entire core
Zoom in
Entire core
Zoom in
Entire core
Zoom in

Figure S5

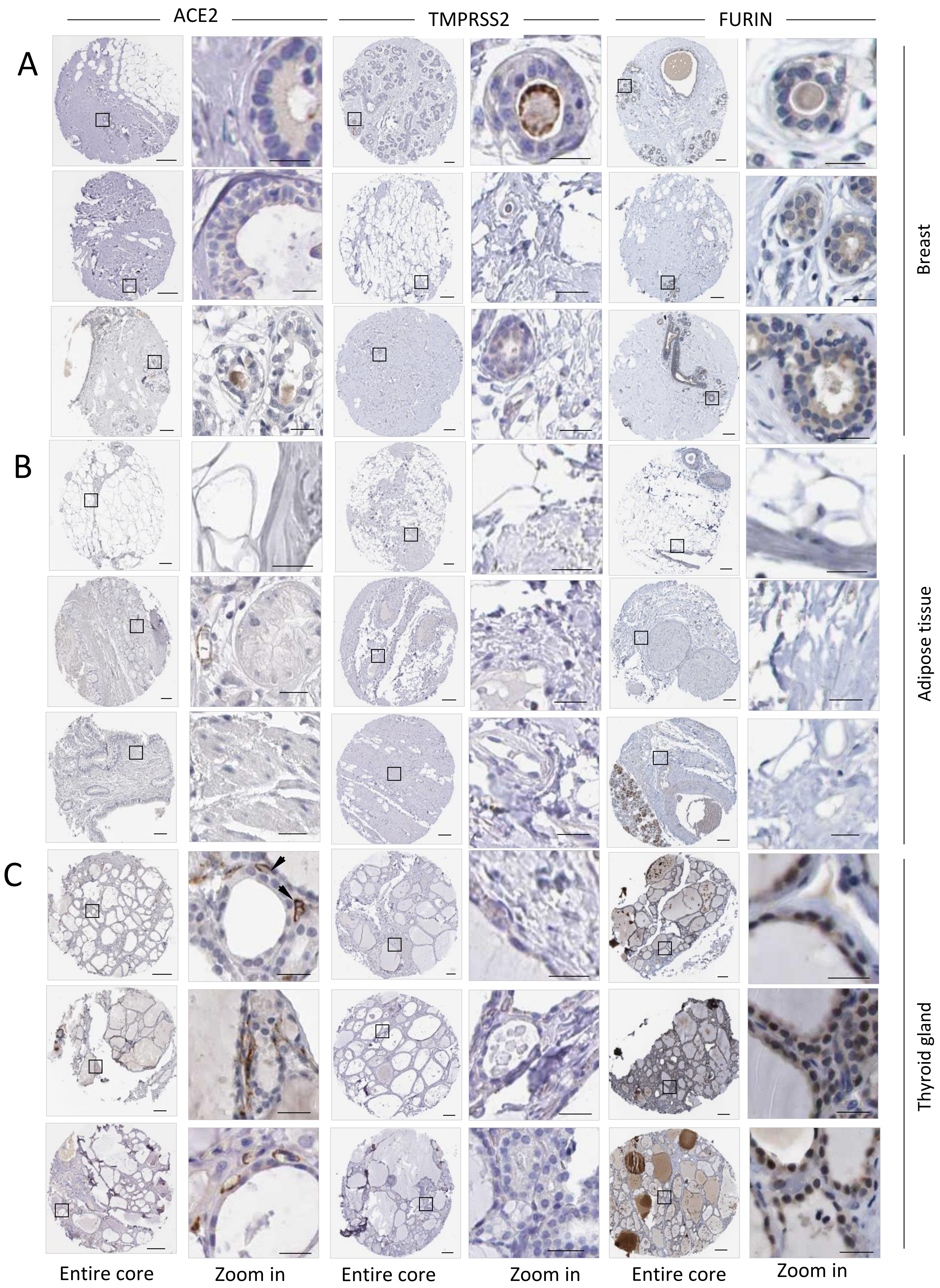
ACE2
TMPRSS2
FURIN
A
B
C
Breast
Adipose tissue
Thyroid gland
Entire core
Zoom in
Entire core
Zoom in
Entire core
Zoom in

Figure S6

G

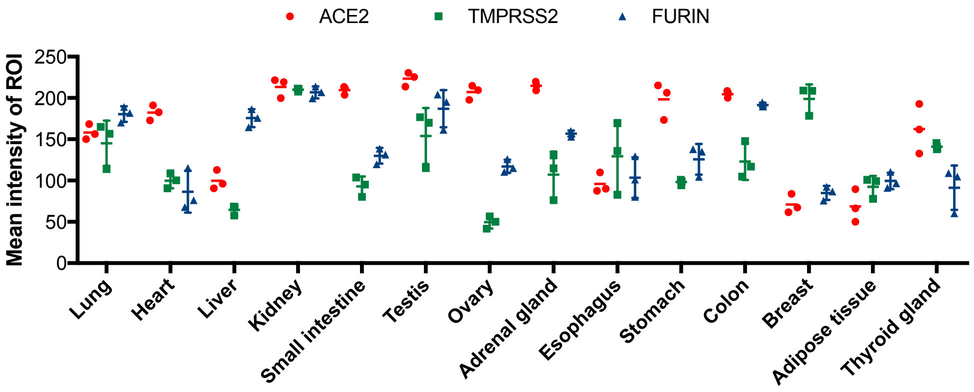
ACE2
TMPRSS2
FURIN
Mean intensity of ROI
250
200
150
100
50
0
Lung
Heart
Liver
Kidney
Small intestine
Testis
Ovary
Adrenal gland
Esophagus
Stomach
Colon
Breast
Adipose tissue
Thyroid gland

Figure S7

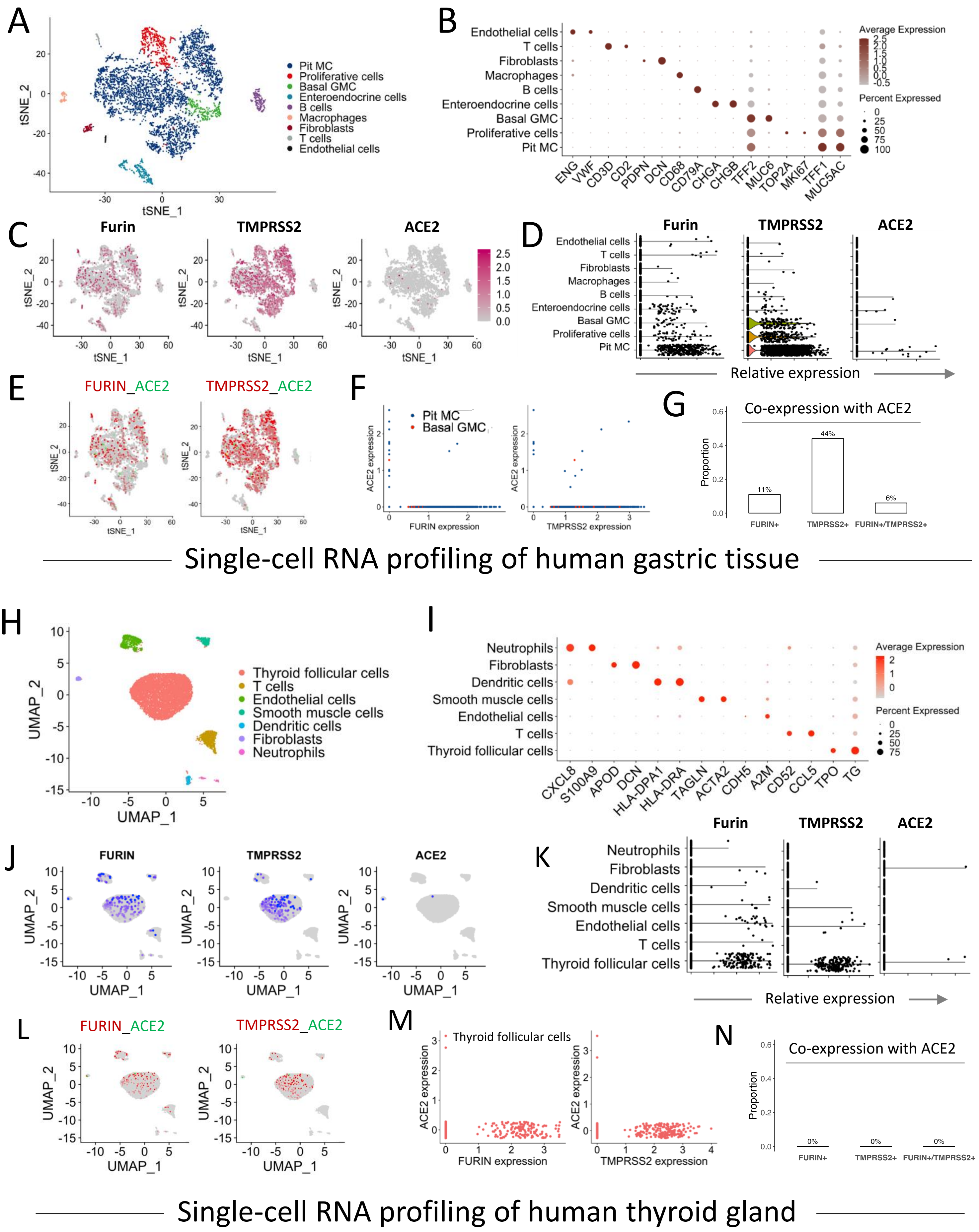
A
Pit MC
Proliferative cells
Basal GMC
Enteroendocrine cells
B cells
Macrophages
Fibroblasts
T cells
Endothelial cells
tSNE_1
tSNE_2
B
Endothelial cells
T cells
Fibroblasts
Macrophages
B cells
Enteroendocrine cells
Basal GMC
Proliferative cells
Pit MC
ENG VWF CD3D CD2 PDPN DCN CD68 CD79A CHGA CHGB TFF2 MUC6 TOP2A MKI67 TFF1 MUC5AC
Average Expression
Percent Expressed
C
Furin
TMPRSS2
ACE2
D
Furin
TMPRSS2
ACE2
Relative expression
E
FURIN_ACE2
TMPRSS2_ACE2
F
Pit MC
Basal GMC
ACE2 expression
FURIN expression
TMPRSS2 expression
G
Co-expression with ACE2
Proportion
11%
44%
6%
FURIN+
TMPRSS2+
FURIN+/TMPRSS2+
Single-cell RNA profiling of human gastric tissue
H
Thyroid follicular cells
T cells
Endothelial cells
Smooth muscle cells
Dendritic cells
Fibroblasts
Neutrophils
UMAP_1
UMAP_2
I
Neutrophils
Fibroblasts
Dendritic cells
Smooth muscle cells
Endothelial cells
T cells
Thyroid follicular cells
CXCL8 S100A9 APOD DCN HLA-DPA1 HLA-DRA TAGLN ACTA2 CDH5 A2M CD52 CCL5 TPO TG
Average Expression
Percent Expressed
J
FURIN
TMPRSS2
ACE2
K
Furin
TMPRSS2
ACE2
Relative expression
L
FURIN_ACE2
TMPRSS2_ACE2
M
Thyroid follicular cells
ACE2 expression
FURIN expression
TMPRSS2 expression
N
Co-expression with ACE2
Proportion
0%
0%
0%
FURIN+
TMPRSS2+
FURIN+/TMPRSS2+
Single-cell RNA profiling of human thyroid gland

Figure S8

Supplementary Table 1. Comparison of RNA and protein levels in different tissues/organs, Related to Figure 1.

Gene ID	Expression level	Lung	Salivary gland	Esophagus	Stomach	Small intestine	Colon	Liver	Pancreas	Thyroid gland	Adrenal gland	Kidney	Urinary bladder	vagina	Skin	Ovary	Fallopian tube	Endometrium	Cervix, uterine	Breast	Testis	Prostate	Cerebral cortex	Cerebellum	Hippocampus	Caudate	Spleen	Heart muscle	Skeletal muscle	Adipose tissue
ACE2	RNA	M	L	H	L	H	H	L	L	H	L	H	N	L	L	H	N	N	N	H	H	N	N	N	N	N	N	H	N	H
	Protein	N	N	N	N	H	L	N	N	L	M	H	N	N	N	N	N	N	N	N	H	N	N	N	N	N	N	N	N	N
TMPRSS2	RNA	H	M	H	H	H	H	M	H	H	N	H	M	M	L	N	N	N	N	H	N	H	N	N	N	N	N	N	N	N
	Protein	N	M	N	M	L	N	N	M	N	N	H	N	N	N	N	N	N	N	N	N	M	N	N	N	N	N	N	N	N
FURIN	RNA	H	H	L	H	H	H	H	H	H	H	H	H	H	H	H	H	H	H	H	M	H	H	H	H	H	H	H	H	H
	Protein	N	H	H	M	M	M	M	H	H	M	H	H	M	N	M	L	M	N	L	M	M	H	H	H	L	L	N	N	N

Note: H: High; M: medium; L: low; N: Negative

Supplementary Table 2. Summary of sample information for single-cell analysis, Related to Figure 3

Organs/tissues	GEO aceesion NO.	Number of samples	Single cell sequencing techology	Number of cells	Mean counts per cell
lung	GSE122960	3	10x Genomics	28819	5421
heart	GSE109816	12	SMART-seq2	8148	69059
liver	GSE115469/ Human Cell Atlas Data Portal	5	10x Genomics	8444	2190
kidney	GSE131685	3	10x Genomics	23366	2270
intestine	GSE125970	6 (ileum:2;colon:2;rectum:2)	10x Genomics	14207	13761
stomach	GSE134520	3	10x Genomics	5281	3249
testis	GSE109037	4	10x Genomics	12829	15174
overy	GSE118127	10	10x Genomics	27857	1990
fetal adrenal gland	GSE134355	1	Mirowell-seq	9809	1495
thyroid	GSE134355	1	Mirowell-seq	8966	880

Supplementary Table 3. Summary of the databases, software and algorithms. Related to Figure 1-5.

RESOURCE	SOURCE
**Databases**	
**Human Protein Atlas**	http://www.proteinatlas.org
**The Genotype-Tissue Expression(GTEx) database**	https://m.gtexportal.org
**Gene Expression Omnibus (GEO) database**	https://www.ncbi.nlm.nih.gov/geo
**Human Cell Atlas Data Portal**	https://data.humancellatlas.org/
**Software and Algorithms**	
**Seurat v3.1.4**	https://github.com/satijalab/seurat
**Harmony v1.0**	https://github.com/immunogenomics/harmony
**clusterProfiler v3.14.3**	https://bioconductor.org/packages/release/bioc/html/clusterProfiler
**R v3.6.1**	https://www.r-project.org/

Supplementary Table 4. Recipe for pH calibration. Related to Figure 6

pH	Pseudo-virus	HCL (0.5 mol/L)	NaOH (2 mol/ L)	DMEM
1.0	200 μl	60 μl	42 μl	98 μl
2.0	200 μl	40 μl	28 μl	132 μl
4.0	200 μl	10 μl	7 μl	183 μl
7.0	200 μl	0 μl	0 μl	200 μl

SUPPLEMENTAL FIGURES LEGENDS

Figure S1. Tissue distribution of ACE2, TMPRSS2 and Furin proteases. Related to Figure 1. (A) the anatomogram of the expression of *ACE2*, *TMPRSS2*, and *FURIN* in male and female human tissues. The colour strength corresponds to the gene expression level. (B-D) The normalized mRNA expression of ACE2, TMPRSS2 and Furin in 61 human tissues and organs from HPA database. (E-G) The protein expression scores for ACE2, TMPRSS2 and Furin in 44 human tissues and organs from HPA database. Note: Figure S1, derived directly from HPA database, is an extension of the data shown in Figure 1, generally with expression information from more tissues.

Figure S2. Replicate images for protein expression of ACE2, TMPRSS2 and Furin *in situ* in tissues of the lung (A), heart (B) and liver (C) shown in Figure 2. **Related to Figure 2.** Generally, 3 IHC images derived directly from HPA database were shown for each tissue. Scale bars: 200 μm for core images and 20 μm for zoom in images.

Figure S3. Replicate images for protein expression of ACE2, TMPRSS2 and Furin *in situ* in tissues of the kidney (A), intestine (B) and testis (C) shown in Figure 2. **Related to Figure 2.** Generally, 3 IHC images were shown for each tissue, except for Furin expression in testis, where only 2 images are available in the HPA database. Scale bars: 200 μm for core images and 20 μm for zoom in images.

Figure S4. Replicate images for protein expression of ACE2, TMPRSS2 and Furin *in situ* in tissues of the ovary (A) and adrenal gland (B) shown in Figure 2. **Related to Figure 2.** Generally, 3 IHC images derived directly from HPA database were shown for each tissue. Scale bars: 200 μm for core images and 20 μm for zoom in images.

Figure S5. Protein expression of ACE2, TMPRSS2 and Furin *in situ* in tissues of the oesophagus (A), stomach (B) and colon (C). **Related to Figure 2.** Generally, 3 IHC images were shown for each tissue, except for TMPRSS2 expression in the colon, where only 2 images are available in the HPA database. Scale bars: 200 μm for core images and 20 μm for zoom in images.

Figure S6. Protein expression of ACE2, TMPRSS2 and Furin *in situ* in tissues of the breast (A), adipose tissue (B) and thyroid gland (C). **Related to Figure 2.** Generally, 3 IHC images derived directly from HPA database were shown for each tissue. Scale bars: 200 μm for core images and 20 μm for zoom in images.

Figure S7. Quantitative analysis for the intensity of protein expression in IHC images. Related to Figure 2

Figure S8. Single cell transcriptomic profiling of ACE2, TMPRSS2 and Furin expression in the gastric tissue and the thyroid gland. **Related to Figure 3.** (A) tSNE plot showing different cell types in the stomach. (B) Dot plot showing the expression level of specific cell markers in each cell types of the gastric tissue. (C) tSNE plot revealing the expression distribution of Furin, TMPRSS2 and ACE2 in the stomach. (D) Violin plot showing the expression level of Furin, TMPRSS2 and ACE2 in different cell types of the stomach. (E-F) Characterization of the co-expression feature of ACE2, Furin and TMPRSS2 in the stomach. (G) Barplot shows the proportion of ACE2 positive cells expressing either or both FURIN and TMPRSS2 in the stomach. (H) UMAP plot representing different cell types in the thyroid gland. (I) Dot plot showing the expression level of known cell markers in each cell type of the thyroid gland. (J) UMAP plot illustrating the expression distribution of Furin, TMPRSS2 and ACE2 in the thyroid gland. (K) Violin plot showing the expression level of Furin, TMPRSS2 and ACE2 in different cell types of the thyroid gland. (L-M) Characterization of the co-expression feature of ACE2, Furin and TMPRSS2 in the thyroid gland. (N) Barplot shows the proportion of ACE2 positive cells expressing either or both FURIN and TMPRSS2 in the thyroid gland. MC: mucous cells and GMC: gland mucous cells.

Transparent Methods

Data Acquisition

For scRNA profiling, the raw gene expression matrices for single cells were downloaded from Gene Expression Omnibus (GEO) database(https://www.ncbi.nlm.nih.gov/geo/) and Human Cell Atlas Data Portal (https://data.humancellatlas.org/). In total, we acquired the single cell gene expression datasets of various normal tissues and organs, including the lung, heart, liver, kidney, intestine, stomach, testis, ovary, adrenal gland and thyroid. Apart from the heart dataset, which was obtained by the smart-seq2 method, a majority of the single cell data were generated through the 10x Chromium platform. The GEO accession number of these datasets is GSE122960 (lung) (Reyfman et al., 2019), GSE109816 (heart) (Wang et al., 2020a), GSE131685 (kidney) (Liao et al., 2020), GSE125970 (intestine) (Wang et al., 2020b), GSE134520 (stomach) (Zhang et al., 2019a), GSE109037 (testis) (Hermann et al., 2018), GSE134355 (thyroid and adrenal gland)(Han et al., 2020), and GSE118127 (ovary) (Fan et al., 2019). The liver dataset was acquired from the Human Cell Atlas Data Portal (MacParland et al., 2018). The summary of sample information for single-cell data was shown in the supplementary table 1.

For tissue distribution of mRNA and protein expression profiles, data were obtained for target genes from the "TISSUE" categories of "THE HUMAN PROTEIN ATLAS" (http://www.proteinatlas.org/) (Uhlen et al., 2015). The mRNA expression from the Genotype-Tissue Expression (GTEx) database was chosen for demonstration with reference to the normalized consensus dataset (Carithers and Moore, 2015). The protein expression scores were extracted directly from the "PROTEIN EXPRESSION SUMMARY" subcategory from the Human Protein Atlas (HPA) database. Immunohistochemistry (IHC) staining was performed on normal human tissue samples (Thul et al., 2017). ACE2 expression was primarily detected with the rabbit antibody (1:250, HPA000288, Sigma-Aldrich) and confirmed with the mouse antibody (1:5000, CAB026174, R&D Systems); TMPRSS2 expression was detected with the rabbit antibody (1:300, HPA035787, Sigma-Aldrich); Furin expression was detected with the rabbit antibody (1:125, sc-20801, Santa Cruz Biotechnology). The information on expression intensity and cell types expressing respective genes was extracted from the staining reports for each staining in the HPA database, where the protein expression scores are based on a best estimate of the "true" protein expression from a knowledge-based annotation. For proteins where more than one antibody has been used, a collective score is set displaying the estimated true protein expression. In order to quantify the ACE2 protein expression level of IHC images, we utilized the commercial Nikon software (NIS elements 4.5) to analyze the mean intensity of positive area. Specifically, we firstly selected the positive area where the original intensities of IHC results were highest, where the intensity of 3 selected areas were measured by the Nikon software. Data are presented as the plot of mean intensity.

Quality Control and Data Normalization

The raw count matrices of single-cell transcriptome were imported into R (version 3.6.1, https://www.r-project.org/) and processed by the Seurat R package(version3.1.4) (Stuart et al., 2019). The filter criteria for low-quality cells were determined on the basis of the number of genes and the percentage of mitochondrial genes in the distinct tissue samples. Generally, cells with less than 200 detected genes, higher than 2500 detected genes and higher than 25% mitochondrial genome transcript ratio were removed. In specific, cells with higher than 72% mitochondrial genome transcript ratio in the heart dataset and cells with higher than 50% mitochondrial genome transcript ratio in the liver dataset were removed as mentioned by the corresponding authors (Wang et al., 2020a, MacParland et al., 2018). Then, the gene expression matrices were normalized and scaled. Briefly, for each cell, the expression counts for each gene were divided by the sum of counts for all genes of that cell, multiplied by a scaling factor (10,000) and log transformed using the "NormalizeData" function in the R package Seurat. Furthermore, 2000 highly variable genes were selected based on a variance stabilizing transformation method for downstream analysis.

Data Integration and Dimension Reduction

To avoid batch effects among samples and experiments in the heart and intestine datasets, we adopted the anchoring procedure in the Seurat R package to integrate the datasets as described previously (Stuart et al., 2019). In brief, the "anchor" correspondences between datasets were identified using the "FindIntegrationAnchors" function with default parameters in Seurat. Then, we used the "IntegrateData" function in Seurat to obtain the batch-corrected expression matrices. We also applied another integration approach called "Harmony" (Korsunsky et al., 2019) to the kidney and testis datasets. The Harmony R package (version1.0, https://github.com/immunogenomics/Harmony) focuses on scalable integration of the scRNA-seq data for batch correction.

In order to reduce the number of dimensions representing each cell, the "RunPCA" function in Seurat was performed to calculate principal components (PCs). Then, the number of PCs used in downstream analysis and visualization was determined based on the "JackStraw" procedure and the elbow of a scree plot. Nonlinear dimensionality reduction algorithms, including uniform manifold approximation and projection (UMAP) and t-distributed stochastic neighbour embedding (t-SNE), were also used to conduct unsupervised clustering of single cells. Specifically, we made use of the UMAP and t-SNE to place cells with similar local neighbourhoods based on the statistically significant PCs to visualize the datasets.

Cell Clustering, Annotation and Visualization

We applied a graph-based clustering approach to assemble cells. Briefly, we constructed a K-nearest neighbour graph based on the Euclidean distance in principal component analysis (PCA) space and refined the edge weights between any two cells based on the shared overlap in their local neighbourhoods through the "FindNeighbors" function in Seurat. Subsequently, we applied the Louvain algorithm using the "FindCluster" function in Seurat to group cells.

We applied two strategies to determine cell types. First, canonical marker genes were applied to annotate each cluster into known biological cell types. Expression dot plots or heatmaps of the marker genes for each cluster are shown in Figure 3. Second, we used the "FindAllMarkers" function from R package Seurat to find differentially expressed genes by comparing each cluster of cells with all other cells. Differentially expressed genes in each cluster were enriched to the cell markers gene sets(Zhang et al., 2019b) using the "clusterProfiler" R package (version 3.14.3) (Yu et al., 2012) to predict the probable cell types.

Umap plots, heatmaps, violin plots, and dot plots were generated using the R package Seurat. In order to visualize co-expression of two genes simultaneously, we adopted the "FeaturePlot" function and "FeatureScatter" function in Seurat.

Cell culture, constructs and pseudovirus production

The HEK293T, 293T-ACE2 and Hela-ACE2 cells were maintained in DMEM (MACGENE Tech Ltd., Beijing, China) supplemented with 10% fetal bovine serum (Kang Yuan Biol, Tianjin, China) and 1% Penicillin-Streptomycin (MACGENE Tech Ltd., Beijing, China). All cells were incubated with 5% CO2 at 37°C. The codon-optimized SARS-CoV-2 S cDNA was synthesized at Genscript Biotech Corporation (Nanjing, China). The wild type S genes of SARS-CoV-2 were cloned into pSecTag2-Hygro-A through seamless homologous recombination.

For the production of MSV-based SARS-CoV-2 pseudotypes, HEK293T cells were co-transfected with an S encoding-plasmid, a Gag-Pol packaging construct (Addgene，8449，USA) and the pQCXIP retroviral vector (Clontech, USA) expressing a luciferase reporter by using Lipofectamine LTX and Plus Reagent (Invitrogen, 1784283, USA) according to the manufacturer's instructions. Cells were incubated for 6 hours at 37 °C in transfection medium, then DMEM containing 10% FBS and cultured for additional 48 hours. The supernatants were harvested and filtered through 0.45 μm membranes and stored at -80 °C.

Pseudovirus assay

Before infection, a defined amount of supernatant containing pseudo-viruses were mixed with certain volumes of hydrochloric acid stock buffer (0.5 M) to produce virus fluids of different pH (1, 2, 4 or 7, respectively). The mixture experiments were performed in triplicates, which gave consistent final pH values with a variation range within 0.1 pH units. Virus mixtures were incubated at room temperature for 15 minutes, after which they were neutralized by 2 M NaOH solution and laid at room temperature for additional 15 minutes. All pH values were determined by a PH meter (FiveEasy™plus, Mettler Toledo, Switzerland). Please find detail recipe for pH calibration in Supplementary Table 3.

293T-ACE2 cells and Hela-ACE2 cells were cultured in DMEM medium supplemented with 10% FBS and 1% PenStrep. The viral fluids were mixed with normal culture media containing 0.5 × 10^4 cells and plated into a 96-well plate with a final volume of 100 ul per well. At the time points of 24 hours, or 48 hours post infection, 100 µL One-Glo-EX (Promega, E6120) was added into the cells for incubation in the dark for 10 min prior to reading on an Enspire 2300 multilable reader (Perkin Elmer, USA). Measurements were done at least in triplicates and relative luciferase units (RLU) were plotted.

Reference

CARITHERS, L. J. & MOORE, H. M. (2015). The Genotype-Tissue Expression (GTEx) Project. Biopreserv Biobank*,* 13**,** 307-308.

FAN, X., BIALECKA, M., MOUSTAKAS, I., LAM, E., TORRENS-JUANEDA, V., BORGGREVEN, N. V., TROUW, L., LOUWE, L. A., PILGRAM, G. S. K., MEI, H., et al. (2019). Single-cell reconstruction of follicular remodeling in the human adult ovary. Nature Communications*,* 10.

HAN, X., ZHOU, Z., FEI, L., SUN, H., WANG, R., CHEN, Y., CHEN, H., WANG, J., TANG, H., GE, W., et al. (2020). Construction of a human cell landscape at single-cell level. Nature.

HERMANN, B. P., CHENG, K., SINGH, A., ROA-DE LA CRUZ, L., MUTOJI, K. N., CHEN, I. C., GILDERSLEEVE, H., LEHLE, J. D., MAYO, M., WESTERNSTRÖER, B., et al. (2018). The Mammalian Spermatogenesis Single-Cell Transcriptome, from Spermatogonial Stem Cells to Spermatids. Cell Reports*,* 25**,** 1650-1667.e1658.

KORSUNSKY, I., MILLARD, N., FAN, J., SLOWIKOWSKI, K., ZHANG, F., WEI, K., BAGLAENKO, Y., BRENNER, M., LOH, P.-R. & RAYCHAUDHURI, S. (2019). Fast, sensitive and accurate integration of single-cell data with Harmony. Nature Methods*,* 16**,** 1289-1296.

LIAO, J., YU, Z., CHEN, Y., BAO, M., ZOU, C., ZHANG, H., LIU, D., LI, T., ZHANG, Q., LI, J., et al. (2020). Single-cell RNA sequencing of human kidney. Scientific Data*,* 7.

MACPARLAND, S. A., LIU, J. C., MA, X.-Z., INNES, B. T., BARTCZAK, A. M., GAGE, B. K., MANUEL, J., KHUU, N., ECHEVERRI, J., LINARES, I., et al. (2018). Single cell RNA sequencing of human liver reveals distinct intrahepatic macrophage populations. Nature Communications*,* 9.

REYFMAN, P. A., WALTER, J. M., JOSHI, N., ANEKALLA, K. R., MCQUATTIE-PIMENTEL, A. C., CHIU, S., FERNANDEZ, R., AKBARPOUR, M., CHEN, C. I., REN, Z., et al. (2019). Single-Cell Transcriptomic Analysis of Human Lung Provides Insights into the Pathobiology of Pulmonary Fibrosis. Am J Respir Crit Care Med*,* 199**,** 1517-1536.

STUART, T., BUTLER, A., HOFFMAN, P., HAFEMEISTER, C., PAPALEXI, E., MAUCK, W. M., HAO, Y., STOECKIUS, M., SMIBERT, P. & SATIJA, R. (2019). Comprehensive Integration of Single-Cell Data. Cell*,* 177**,** 1888-1902.e1821.

THUL, P. J., AKESSON, L., WIKING, M., MAHDESSIAN, D., GELADAKI, A., AIT BLAL, H., ALM, T., ASPLUND, A., BJORK, L., BRECKELS, L. M., et al. (2017). A subcellular map of the human proteome. Science*,* 356.

UHLEN, M., FAGERBERG, L., HALLSTROM, B. M., LINDSKOG, C., OKSVOLD, P., MARDINOGLU, A., SIVERTSSON, A., KAMPF, C., SJOSTEDT, E., ASPLUND, A., et al. (2015). Proteomics. Tissue-based map of the human proteome. Science*,* 347**,** 1260419.

WANG, L., YU, P., ZHOU, B., SONG, J., LI, Z., ZHANG, M., GUO, G., WANG, Y., CHEN, X., HAN, L., et al. (2020a). Single-cell reconstruction of the adult human heart during heart failure and recovery reveals the cellular landscape underlying cardiac function. Nature Cell Biology*,* 22**,** 108-119.

WANG, Y., SONG, W., WANG, J., WANG, T., XIONG, X., QI, Z., FU, W., YANG, X. & CHEN, Y. G. (2020b). Single-cell transcriptome analysis reveals differential nutrient absorption functions in human intestine. J Exp Med*,* 217.

YU, G., WANG, L. G., HAN, Y. & HE, Q. Y. (2012). clusterProfiler: an R package for comparing biological themes among gene clusters. OMICS*,* 16**,** 284-287.

ZHANG, P., YANG, M., ZHANG, Y., XIAO, S., LAI, X., TAN, A., DU, S. & LI, S. (2019a). Dissecting the Single-Cell Transcriptome Network Underlying Gastric Premalignant Lesions and Early Gastric Cancer. Cell Reports*,* 27**,** 1934-1947.e1935.

ZHANG, X., LAN, Y., XU, J., QUAN, F., ZHAO, E., DENG, C., LUO, T., XU, L., LIAO, G., YAN, M., et al. (2019b). CellMarker: a manually curated resource of cell markers in human and mouse. Nucleic acids research*,* 47**,** D721-D728.
